# Graphene oxide-modified PEEK composites: Properties and applications in orthopaedic repair — A review

**DOI:** 10.1016/j.jot.2025.11.004

**Published:** 2025-12-23

**Authors:** Mingjing Zhang, Shuzhong Liu, Jinyi Xing, An Song, Liqi Ng, Nan Tao, Xin Su, Changning Sun, Chaozong Liu

**Affiliations:** a Institute of Orthopaedic and Musculoskeletal Science, University College London, Royal National Orthopaedic Hospital, London, HA7 4LP, UK; b Department of Orthopaedic Surgery, Peking Union Medical College Hospital, Peking Union Medical College and Chinese Academy of Medical Sciences, Beijing, 100730, China; c Department of Endocrinology, National Health Commission Key Laboratory of Endocrinology, State Key Laboratory of Complex Severe and Rare Diseases, Translational Medicine Center, Peking Union Medical College Hospital, Chinese Academy of Medical Sciences & Peking Union Medical College, Beijing, 100730, China; d State Key Laboratory for Manufacturing System Engineering, School of Mechanical Engineering, Xi'an Jiaotong University, Xi'an, 710049, China

**Keywords:** Bone repair, Clinical translation, Functionalization, Graphene oxide (GO), Graphene oxide-modified PEEK (GO-PEEK), Polyetheretherketone (PEEK)

## Abstract

Critical-sized bone defect repair remains a major challenge in orthopaedics and tissue engineering. Polyetheretherketone (PEEK) has attracted wide attention due to its excellent mechanical compatibility and radiological transparency; however, its inherent bioinertness and insufficient antibacterial properties restrict its clinical utility. In recent years, the incorporation of graphene oxide (GO) has markedly improved the biological performance of PEEK. GO can increase surface hydrophilicity and roughness, enhance protein/ion adsorption, and promote osteoblast adhesion and differentiation, while simultaneously strengthening antibacterial and immunomodulatory effects without compromising, and in some cases even enhancing, mechanical performance. In vitro studies demonstrate that GO-PEEK stimulates osteogenic gene expression and mineralized nodule formation, while in vivo animal models confirm superior osseointegration and new bone formation compared with controls. Synergistic modifications, such as combination with hydroxyapatite, metallic ions, or antimicrobial peptides, further amplify both osteogenic and antibacterial outcomes. Nevertheless, clinical translation of GO-PEEK remains hampered by challenges including long-term stability, potential particulate-related risks, the dynamic balance between antibacterial and osteogenic functions, and issues of manufacturing scalability, consistency, and sterilization compatibility. Future research should focus on establishing a “structure–property–safety” design paradigm, developing temporally programmed multifunctional strategies, and advancing 3D-printed personalized fabrication, with low-load applications such as alveolar or cranial bone repair as potential pioneer indications. Overall, GO-PEEK composites exhibit significant promise in contexts such as post-tumour bone reconstruction, dental implantation, and spinal or joint implants, and are expected to achieve successful clinical translation under evidence-based validation and standardised manufacturing pathways.

***The Translational Potential of this Article***: The findings of this review highlight the potential of graphene oxide-modified PEEK (GO-PEEK) composites as next-generation orthopaedic biomaterials. By integrating enhanced osteogenic activity, antibacterial efficacy, and immunomodulatory capacity into a mechanically compatible and radiolucent polymer, GO-PEEK offers a multifunctional platform for bone repair. Importantly, its promising performance in vitro and in vivo provides a foundation for translation into clinical contexts such as dental implants, spinal fusion cages, and tumour-related bone defect reconstruction. Addressing challenges in long-term stability, sterilization compatibility, and large-scale manufacturing will be critical to establish a clear regulatory and translational pathway from laboratory research to clinical practice.

## Graphical abstract

**Figure:**
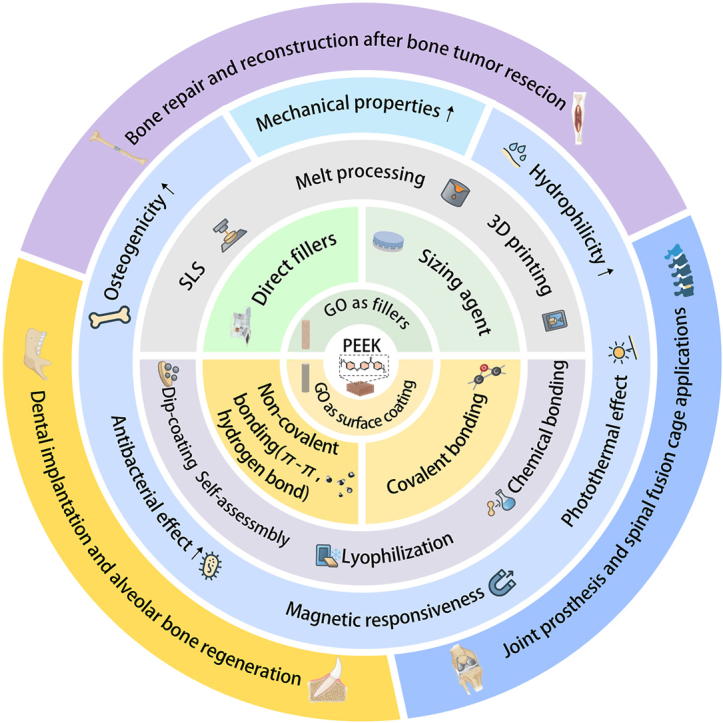

## 1 Introduction

Bone defects (BDs) refer to the loss or destruction of normal bone tissue, which may result from various etiologies, including trauma, osteoporosis, osteomyelitis, osteonecrosis, and bone tumors, with a wide range of severity [1,2]. When the extent of bone loss exceeds the body's inherent regenerative capacity, the defect is classified as a critical-size bone defect (CBD), which typically requires surgical intervention to facilitate effective bone regeneration [3]. Bone defects are not only common but also clinically significant, often resulting in long-term functional impairment and a marked decline in patients' quality of life [4,5]. This condition places substantial demands on clinical management and contributes to a growing socioeconomic burden. Despite considerable advances in bone repair strategies in recent years, the successful reconstruction of CBDs remains a major clinical and translational challenge [6,7]. BDs are not only highly prevalent but also clinically significant, often resulting in long-term functional impairment and a markedly reduced quality of life for affected patients [4,5].

Bone regeneration is a highly orchestrated and multifaceted pathophysiological process, encompassing a series of interdependent events such as osteogenesis, osteoclast-mediated bone resorption and remodeling, apoptosis, osteoblastic differentiation, biomineralization, angiogenesis, and immune regulation [8]. Autologous bone grafting remains the clinical gold standard for the treatment of bone defects. However, its application is constrained by limited donor availability and the risk of donor site morbidity, which can increase surgical complexity and impose additional physical and financial burdens on patients [9]. Allogeneic bone grafts can partly compensate for the scarcity of autologous sources, yet they carry inherent risks of immunogenic rejection and potential disease transmission. Additionally, the irregular morphology of many bone defects further complicates the use of conventional grafting approaches. In the absence of timely and effective intervention, patients are at increased risk of developing complications such as chronic pain, pathological fractures, infections, and non-union—conditions that severely compromise both physical function and quality of life [10]. Consequently, the development of safer, more effective artificial bone substitutes has become a key focus in regenerative medicine and orthopaedic research. Compared to autografts and allografts, synthetic bone graft materials offer numerous advantages, including scalable production, diverse raw material sources, cost-effectiveness, and avoidance of donor site morbidity [11]. More importantly, recent advancements in materials science and tissue engineering have substantially improved the mechanical properties, bioactivity, and customizability of synthetic bone scaffolds, opening new avenues for the repair and reconstruction of complex bone defects.

**Fig. 1:**
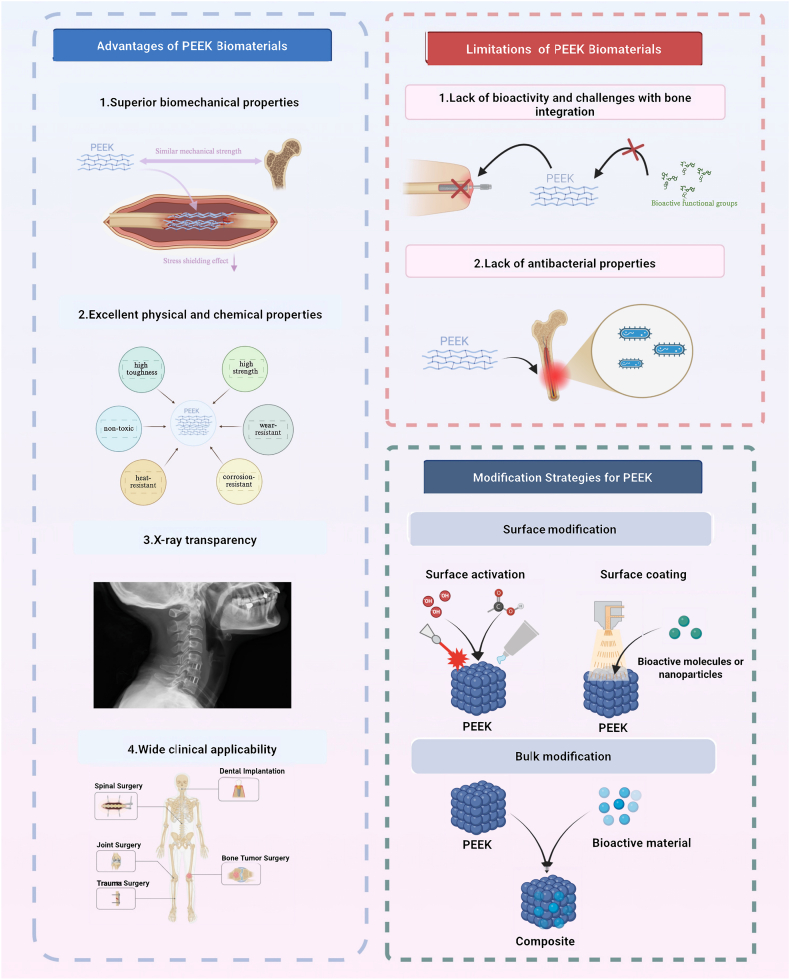
**Advantages, limitations, and modification strategies of PEEK biomaterials.** PEEK exhibits superior biomechanical compatibility, excellent physicochemical stability, X-ray transparency, and wide clinical applicability. However, its poor bioactivity and lack of antibacterial properties limit bone integration. Current modification strategies include surface activation, surface coating, and bulk modification with bioactive materials to enhance interfacial bonding and biological performance. Schematics created with BioRender.

An ideal bone implant material should exhibit mechanical properties that are well-matched to those of native bone, along with favorable bioactivity to support and enhance bone regeneration [10,11]. Currently, synthetic bone graft materials commonly fall into three main categories: (A)Metallic materials (e.g., stainless steel, titanium and its alloy Ti-6Al-4V, cobalt-chromium alloys), which provide excellent mechanical strength and corrosion resistance, making them suitable for structural support at defect sites. However, their elastic moduli are substantially higher than that of natural bone, often resulting in stress shielding and subsequent secondary bone resorption [[10], [11], [12]]. (B)Ceramic materials (e.g., hydroxyapatite, tricalcium phosphate), which demonstrate high bioactivity and osteoconductivity but are limited by their intrinsic brittleness and poor fracture toughness. (C)Polymeric materials (e.g., polylactic acid [PLA], polycaprolactone [PCL]), which offer desirable features such as biodegradability, biocompatibility, and ease of processing. Nevertheless, their insufficient mechanical strength restricts their application in load-bearing bone defect repair [[13], [14], [15], [16], [17]]. Despite these advances, limitations such as inadequate immunocompatibility and suboptimal biological performance continue to impede the broader clinical translation of these materials. Consequently, the design and development of bone repair materials that combine appropriate mechanical strength, excellent biocompatibility, and tunable biological functionalities have emerged as a key priority in orthopaedic biomaterials research (Fig. 1).

Polyetheretherketone (PEEK) is a high-performance thermoplastic polymer that has garnered significant interest in the orthopaedic field due to its favorable physicochemical and mechanical properties, including excellent biocompatibility, high thermal and chemical stability, corrosion resistance, adjustable elastic modulus, intrinsic self-lubrication, ease of processing, and radiolucency [[18], [19], [20], [21]]. In contrast to conventional metallic implants, PEEK exhibits an elastic modulus more closely approximating that of native cortical bone, thereby reducing stress shielding and enhancing the mechanical compatibility at the bone–implant interface, which facilitates superior osseointegration [[22], [23], [24], [25]]. In addition, PEEK demonstrates outstanding imaging compatibility, producing minimal artifacts in both magnetic resonance imaging (MRI) and computed tomography (CT), thus enabling accurate postoperative assessment and monitoring [26,27]. Following its approval by the U.S. Food and Drug Administration (FDA) in 2013, PEEK-based implants have been widely adopted in various orthopaedic subspecialties, including spinal surgery, joint arthroplasty, trauma reconstruction, and orthopaedic oncology [[28], [29], [30], [31], [32], [33], [34]](Fig. 1).

Despite its numerous advantages, PEEK's inherent bioinertness and poor surface hydrophilicity significantly limit its widespread application in bone regeneration [35,36]. The lack of intrinsic functional groups that facilitate osteogenesis or antimicrobial activity results in suboptimal osseointegration and limited bioactivity [[37], [38], [39], [40], [41]]. To overcome these limitations, various modification strategies have been explored, including: (A) the fabrication of micro- and nano-scale surface structures via techniques such as sandblasting and sulfuric acid etching, which improve cell adhesion and enhance tissue anchorage([42], [43], [44], [45], [46]). (B) the incorporation of bioactive agents or functional polymers through surface coating, chemical grafting, or bulk blending methods. Common additives include titanium dioxide (TiO_2_) and hydroxyapatite (HAp), which have demonstrated promising osteoinductive potential [18,19,[47], [48], [49]]. Although these approaches have contributed to enhanced biological performance, challenges remain in achieving robust osteoinductivity, sustained antimicrobial activity, and integrated multifunctionality (Fig. 1).

In recent years, the integration of graphene and its derivatives has opened new avenues for the functionalization of orthopaedic implant materials. Among these, graphene oxide (GO) has attracted particular interest owing to its high density of oxygen-containing functional groups—such as carboxyl, hydroxyl, carbonyl, and epoxy—which impart excellent aqueous dispersibility and enable the modulation of cellular behavio [50,51]. GO-based materials have shown considerable promise in diverse biomedical applications, including drug delivery, antimicrobial therapy, cancer treatment, and tissue engineering, and are increasingly being explored as emerging candidates for bone repair strategies [13,16]. An ideal bone repair material must combine several key properties, including biocompatibility, osteoinductivity, antimicrobial efficacy, and mechanical compatibility. The incorporation of GO and other functional nanomaterials into PEEK scaffolds represents a compelling strategy to enhance the biological performance of the composite, while simultaneously conferring multifunctional capabilities such as antibacterial and anticancer activity [[52], [53], [54], [55], [56]].

In summary, the multifunctional characteristics of graphene oxide (GO) combined with the superior mechanical properties of polyetheretherketone (PEEK) offer a novel approach for the development of composite biomaterials exhibiting enhanced osseointegration, osteogenic activity, antibacterial efficacy, and anticancer functionality. Although significant challenges remain in further improving the biological performance of PEEK while preserving its inherent mechanical advantages, research on GO-PEEK composites has increasingly become a forefront and hotspot in the field of bone regeneration. This review systematically summarizes recent advances in GO-modified PEEK composites, focusing on their feasibility, underlying mechanisms, and future prospects in bone tissue repair.

## 2 Preparation methods of GO-PEEK composites

### 2.1 Preparation strategies

**Fig. 2:**
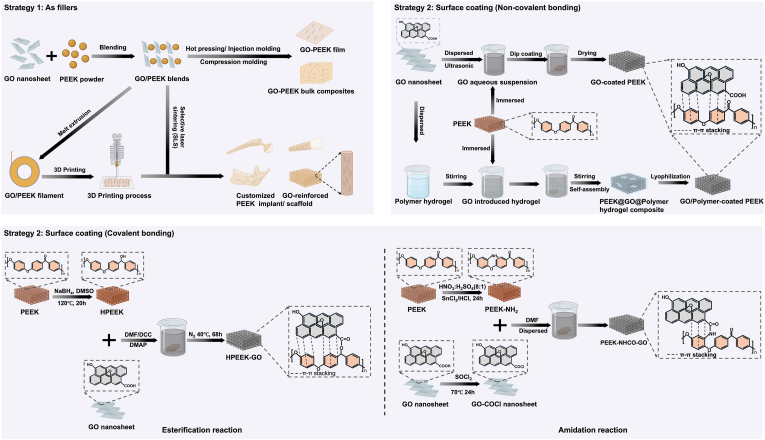
**Preparation strategies for GO/PEEK composites**. Schematics created with BioRender. Strategy 1: GO nanosheets used as fillers are incorporated into the PEEK matrix via blending, hot pressing, injection molding, or 3D printing to form bulk composites or customized implants/scaffolds. Strategy 2: GO applied as surface coatings enhances interfacial properties and bioactivity. (a) Non-covalent bonding via π–π stacking and hydrogen bonding. (b) Covalent bonding through esterification or amidation reactions, yielding strong and stable interfacial adhesion.

With the rapid progress of materials science, the integration of GO into PEEK has emerged as a promising approach for developing next-generation composites. Preparation strategies can be broadly divided into two categories. As a reinforcing phase, GO can be uniformly dispersed within the PEEK matrix to enhance mechanical strength and thermal stability [[57], [58], [59], [60], [61], [62], [63], [64], [65], [66], [67], [68], [69], [70], [71], [72], [73], [74], [75], [76], [77]]. Alternatively, it can serve as an interfacial enhancer by grafting onto carbon fibers (CF) or carbon nanotubes (CNTs) before incorporation, thereby strengthening interfacial bonding and load transfer. This approach markedly improves the mechanical and fatigue properties of the composites, making them particularly suitable for load-bearing orthopaedic implants ([66], [67], [68], [69], [70], [71], 77, 78). As a surface coating, GO improves the hydrophilicity and bioactivity of PEEK. The underlying interactions include non-covalent forces (π–π stacking, hydrogen bonding) ([79], [80], [81], [82], [83], [84], [85], [86], [87], [88], [89], [90], [91], [92], [93], [94], [95]). and covalent linkages via esterification or amidation reactions ([96], [97], [98], [99]). with covalent bonding providing superior adhesion and long-term stability (Fig. 2).

### 2.2 Fabrication techniques and their advantages and limitations

For bone repair applications, the fabrication of GO-PEEK composites can be broadly divided into two categories: blending techniques and surface coating techniques, each with specific merits and constraints.

#### 2.2.1 Blending techniques

These include hot pressing [[67], [68], [69], [70]], melt extrusion and injection molding [[71], [72], [73], [74]], and selective laser sintering (SLS)/3D printing [75,76,[100], [101], [102]], Compression molding [101,103,104]. Melt extrusion and injection molding provide excellent reproducibility and industrial scalability; however, the elevated processing temperatures may damage GO nanosheets, deplete functional groups, or induce aggregation, thereby weakening interfacial reinforcement [105]. SLS/3D printing allows the fabrication of patient-specific, porous structures highly suitable for bone scaffolds [106]. Nevertheless, high equipment cost, narrow processing parameters, and the stringent requirements for GO dispersion and thermal stability remain significant barriers to clinical translation [107](Fig. 2) (Table 1).

**Table 1:** GO combination strategies and their resulting enhancements in the physicochemical properties of PEEK composites.

Combination Strategy		Matrix	Fillers/Coatings Composition	Fabrication Method	Interactions	Effect of GO on Physicochemical Properties	Optimum Composition/Trend	Ref.
**Fillers**	Sizing agents	PEEK	GO: ∼1 wt% MWCNT: 0.5–1 wt%	Hot pressing	π–π stacking	↑Thermal conductivity(0.18 → 0.36 W/m·K); ↑Storage modulus (58.4 → 68.2 %) ↑Crystallinity	1 wt% GO + 0.5 wt% MWCNT; higher loadings → agglomeration and ↓ performance	63
	Direct filler	PEEK	GO: 0–1 wt % c-aminopropyl trimethoxysilane-modified graphene oxide (GO-Si)	Hot pressing	Not specified	↑Wear resistance; ↓ Friction coefficient (lowest at 0.1 wt% GO-Si); ↑Thermal stability; ↑ Crystallinity; smoother worn surface observed	0.1 wt% GO-Si, excessive GO → aggregation and ↑ friction	64
	Sizing agents	PEEK	GO: 0–0.75 wt% s-PSF coated CF	Hot pressing	Not specified	↑Flexural strength (to 847.29 MPa) ↑Flexural modulus (to 63.77 GPa) ↑Interlaminar shear strength(ILSS) (to 73.17 MPa); ↑ Tg (+4 °C); ↑ Density; ↓Water absorption	0.5 wt% GO; higher GO → aggregation → decreased ILSS and flexural strength	65
	Sizing agents	PEEK	GO: ∼1 wt% CF: 30 wt%	Melt extrusion and injection molding	Not specified	↑hydrophilicity(↓ Contact angle (79.9° → 59.5°) vs CF/PEEK)	GOCF/PEEK → highest wettability and bioactivity	66
	Direct filler	PEEK	GO: 0.1-1 wt% TDI–PEK-L	Melt extrusion and injection molding/3D printing	Not specified	↑ Tensile (+6.8 %), ↑ modulus (+7.0 %), ↑ elongation (+31.6 %), ↑ flexural (+7.1 %), ↑ impact (+20.5 %); ↓ friction (−27.3 %) and ↓ wear rate (−18.3 %)	0.1 wt% LFG: optimal mechanical strength and toughness; 0.5 wt% LFG: best tribological (wear and friction) performance >0.5 wt% LFG: aggregation and ↓ ductility	67
	Ssizing agents	PEEK	GO: 0.5–2.0 wt% SiO_2:_ 30 wt%	Melt blending and injection molding	π-π∗ conjugation	↑ Tensile strength (+12.6 %), ↑ modulus (+39.4 %), ↑ hydrophilicity; ↑ thermal stability	1.5 wt% GO → best mechanical & surface performance, ≥2 wt% → GO restacking and reduced performance	68
	Sizing agents	PEEK	GO: ∼1 wt% CF: 30 wt%	Melt extrusion and injection molding	Not specified	↑Flexural strength (+51 %), ↑ Compressive strength (+46 %), ↑ Hardness (+30 %), ↑ Flexural modulus to 11.67 GPa, ↑ Compressive modulus to 6.12 GPa, ↓ ↑Hydrophilicitycontact angle (65.8° vs 73.6°)	1 wt% AGO gave highest mechanical and surface properties tested; no trend beyond 1 wt% reported	69
	Direct filler	PEEK	GO: 0.1–5 wt%	Melt extrusion and injection molding	π-π∗ conjugation	↑Compressive, flexural, and tensile properties, ↑hardness	0.5 wt% GO; >0.5 wt% led to agglomeration and decreased mechanical properties	70
	Direct filler	PEEK	GO: 0–1.25 wt% HAp: 20 wt%	Selectively laser sintering (SLS)	π-π stacking	↑ Compressive strength (+79.45 %); ↑ Modulus (+42.07 %); ↑ Thermal stability; ↓ Agglomeration below 1 wt% GO; Improved HAP dispersion	1 wt% GO; >1 wt% → GO agglomeration ↓ mechanical strength	71
	Direct filler	PEEK and PVA mixture	GO: 0–2 wt%	Selective laser sintering (SLS)	π-π stacking interaction Hydrogen bonds (with PVA)	↑ Compressive strength +97.16 %; ↑ Modulus +147.06 % (1 wt% GO); ↑ Thermal stability; ↑ Hydrophilicity (contact angle ↓ from 85.52° → 78.16°); ↑ Water absorption and degradability; uniform PVA dispersion	1 wt% GO optimum; >1.5 wt% → GO aggregation, defects, mechanical decline	72
	Direct filler	SPEEK	rGO: 0–1.5 wt%; HAp: 3.5–5 wt%	Melt extrusion and 3D printing	Not specified	↑ Compressive strength with ↑ rGO ↑ Thermal stability	1.5 wt% rGO + 3.5 wt% HAp → highest compressive strength & thermal performance	100
	Direct filler	PEEK	GO: 0–1.5 wt%; HAp: 0–30 wt%	Compression molding	π-π stacking	↓ Friction coefficient, ↓ Wear rate (1.60 × 10^−6^ mm^3^/N·m) ↑ Transfer-film stability and surface smoothness (Ra ≈1.36 μm) ↑Dispersion and load transfer	0.5 wt% GO + 10 wt% HAp → lowest wear rate & friction; higher reinforcement → agglomeration, COF↑, wear rate↑	101
	Direct filler	PEEK	GO: 0–0.75 wt%	Mold casting and sintering	Hydrogen bonding	↑ GIC (+109.3 %) ↑ KIC (+35.6 %) ↑ Tensile strength (+14.7 %) ↑ Flexural strength (+5.0 %) ↓ Crystallinity ↑ Td_5_ → thermal stability maintained	GO-CTS = 0.25 wt% → best balance of toughness & strength; Pure GO peaks at 0.5 wt% but less effective	102
	Direct filler	PEEK	GO: 0–1.5 wt% HAp: 0–30 wt%	Compression molding	π-π stacking	GO 0.5 wt% + HA 10 wt%, ↓Coefficient friction (COF), ↓Wear rate, ↑ Hardness	0.5 wt% GO + 10 wt% HAp → lowest wear rate & friction; higher (>1 wt%) GO → agglomeration, COF↑, wear rate↑	103
	Direct filler	PEEK	GO: 0.5 wt% HAp: 10 wt%	Compression molding	π-π stacking	↑ Tensile strength (+118 %) ↑ Elastic strain to 6.95–11.35 % ↑ Hardness (76.4 → 79.6 Hv) ↑ T_m_ from 340 → 351 °C ↓ Surface roughness (Ra 1.62 → 1.36 μm) ↓ COF (0.025–0.141) ↓ Wear rate (3.39 × 10^−6^ → 1.69 × 10^−6^ mm^3^/N·m)	0.5 wt% GO + 10 wt% HA → best mechanical & tribological behavior; higher GO not tested	103
	Direct filler	PEEK	GO: 5 wt% HAp: 0–40 wt%	Mold casting and sintering	Not specified	↑ Crystallinity up to 47.3 % (HAp 20 wt%); ↑ Thermal stability; ↑ Compressive strength (14.2 → 21.45 MPa at HAp 30 %); ↑ hydrophilicity	5 wt% GO + 30 wt% HAp optimum → highest strength (21.45 MPa) >40 wt% HA → agglomeration and strength ↓ 13.95 Mpa	104
	Direct filler	PEEK	rGO: 1–3 wt% HAp: 0–30 wt%	Melt extrusion and 3D printing	Not specified	↑ Compressive modulus (≈+60 %) and strength (≈+50 %) for PEEK-69/cHAp-30/rGO-1; PEEK-87/cHAp-10/rGO-3 → highest stress tolerance (25.32 GPa) and lowest deformation (9.29 mm)	PEEK-87/HAp-10/rGO-3 wt% → best mechanical performance and lowest deformation; higher rGO >3 wt% not studied	155
	Direct filler	PEEK	GO: 1 wt% PDA ZrO_2_: 10 wt%	Melt extrusion and injection molding	π-π stacking	↑ Tensile strength (93.1 → 103.3 MPa) → ↑ Bending strength (↑17.2 %) → ↑ Compressive strength (↑9.8 %) → ↑ Hydrophilicity → Stable photothermal response (ΔT = 40.6 °C under 2 W, 808 nm) → Uniform nanoparticle dispersion	1 wt% GO (PGPZ) → highest strength & photothermal stability	167
**Surface coating**	Surface coating (Non-convalent bonding)	Sulfonated PEEK (SPEEK)	GO: 0.5 wt% SPEEK: 1 wt%	Dip-coating (X5 cycles)	π–π stacking, hydrogen bonding	↑Hydrophilicity(contact angle ↓ from 90° → 47.7°); Surface roughness↑; Wettability↑ Elastic modulus ≈3–4 GPa (unchanged)	0.5 wt% GO–SPEEK → best synergy of antibacterial and osteogenic properties	18
		PEEK	GO: 0.8 mg/mL pDA: 2 mg/mL BFP: 1 mM	Dip-coating (X6 cycles)	π–π stacking	↑Hydrophilicity (contact angle ↓ 76°→25°) Photothermal conversion ΔT ≈ +54 °C (808 nm, 0.5 W cm^−2^), stable for three cycles	GO/pDA/BFP hybrid exhibited highest photothermal efficiency and hydrophilicity without degradation.	73
		SPEEK	GO: 0.75 wt% Bioactive glass	Spin-coating	Not specified	↑ Hydrophilicity (contact angle ↓ 71° → 14.7°); strong adhesion (74.9 MPa); transparent homogeneous coating with ∼7 μm thickness	0.75 wt% GO group exhibited highest hydrophilicity vs control group	74
		SPEEK	GO: 0.1 wt% SPEEK	Dip-coating	π–π stacking and hydrogen bonding	↑ Hydrophilicity (contact angle ↓ 82.7° → 10.9°) ↑ Surface energy; ↑ Surface roughness (3D pores 200–500 nm); ≈ Mechanical strength retained; ↑ Chemical activity	GO–SPEEK → best hydrophilicity and bioactivity; GO > SPEEK > PEEK; stable coating without degradation	75
		SPEEK	ZnO/GO mass ratio 3:1ZnO/GO mass ratio 4:1ZnO/GO mass ratio 5:1	Dip-coating	π–π stacking	↑ Surface roughness (Ra↑), ↑ hydrophilicity (contact angle ↓ from 88.4° to 51.2°)	ZnO:GO = 4:1 showed best antibacterial effect biocompatibility, and hydrophilicity. ZnO/GO mass ratio↑→ surface roughness ↑	76
		PEEK	rGO: 1–3 wt%	Dip-coating	Not specified	↑compressive strength, ↑surface roughness, ↑improved wettability	3 wt% rGO → highest compressive strength, enhanced surface roughness, and improved wettability; ↑rGO → ↑grain density, ↑protein adsorption	77
		PEEK	GO: 0.75 wt% nHAp	Dip-coating	π-π stacking	↓ Surface roughness (Ra = 21 nm vs 54 nm for PEEK); ↑ Hydrophilicity (contact angle ↓ from 71.3° to 54.6°)	GO 0.75 wt% yielded optimal balance of dispersion, adhesion, and biocompatibility	80
		SPEEK	GO: 1 wt% nisin: 0.04 mmol/mL	Dip-coating	Not specified	↑ Hydrophilicity (CA ↓91°→24°) → ↑ O/N content (O 27.7 %, N 9.8 %) → stable GO coverage → porous anchoring	GO + nisin synergy → highest hydrophilicity & stability	81
		SPEEK	GO MnFe_2_O_4_	Hydrothermal synthesis (MFO/GO) → PDA-assisted coating on SP	Not specified	↑ NIR absorption (200–1000 nm) → ↑ photothermal efficiency (45.3 → 56.1 °C, 1 W cm^−2^) → GPx-mimetic GSH oxidation (54 → 67 % depletion under NIR) → ↑ ROS accumulation	SP–P–MFO/GO shows best GSH consumption and photothermal stability	82
			GO: 0.1 wt%	Dip-coating	π-π stacking	↑hydrophilicity (contact angle ↓), ↑roughness, stable GO layer resistant to ultrasonication	CF concentration↑→Surface roughness↑; GO–40CF/PEEK exhibited highest surface roughness,and hydrophilicity	83
		30 wt% CF-reinforced SPEEK	rGO5 wt% HAp:10 wt%	Spin coating	physical embedding and interfacial bonding	↓ Contact angle (112.5°→ 20°/54.5°/47°) → ↑ hydrophilicity; ↑ surface porosity → better coating anchorage; no structural change in PEEK	HAp coating (no rGO) → highest hydrophilicity and bioactivity; rGO addition slightly reduced wettability	87
		SPEEK	GO PEEK PDA	Self-assembly	π–π stacking & H-bonding between PDA and GO, strong interfacial adhesion on PEEK	↑ Hydrophilicity (Contact Angle 105.5°→38.5°) → ↑ protein adsorption; ↑ M2 macrophage polarization (17.6 %→47.4 %); ↓ pro-inflammatory cytokines (IL-1β, IL-18 ↓); ↑ osteogenic gene expression (RUNX2, COL-I ↑)	PAG (PEEK–PDA–GO) showed optimal wettability and surface uniformity	90
		SPEEK	GO: 0.1 wt%	Dip-coating	π-π stacking	↑ Hydrophilicity (contact angle 83.5° → 48.7°)	GO-SPEEK (10 min sulfonation + GO 1 mg/mL) → optimal hydrophilicity	91
		SPEEK	GO: 0.1 wt% Ag^+^: 10 × 10 ^−3^M	Dip-coating	π–π stacking and hydrogen bonding	↑ Surface roughness & porosity (0.5–2 μm) → ↑ hydrophilicity (CA ↓ to ∼63°) → Ag^+^ slow release (47.9 % in 3 days) → durable structure	GO layer (10–25 nm) + Ag ^+^ uniformly dispersed → best stability and antibacterial effect	92
		CF-reinforced SPEEK	GO: 0.1 wt%	Dip-coating	π-π stacking	↑Surface roughness, ↑hydrophilicity; ↑Crystallinity; ↑Bending strength ↑ to ∼265 MPa; ↑Compressive modulus ∼1 GPa ↑ vs. CF/PEEK	GO–SCF/PEEK exhibited best balance between bioactivity and mechanical integrity	94
		PEEK	GO: 0.4 wt%	Spin-coating, dip-coating, plasma stabilization	π-π stacking	↑ Surface roughness → enhanced protein adsorption; ↑ O/C ratio → improved hydrophilicity; ↑ stability → better coating adhesion	Spin-coated GO + CH_4_ + O_2_ plasma yielded uniform, stable, hydrophilic coating; dip-coated GO less stable in wet media	95
		PEEK	GO: 0.05 wt% PDA	Self-assembly + Freexe drying	π–π stacking & covalent bonding between PDA and GO; strong interfacial adhesion on PEEK	↑Hydrophilicity(↓ Water contact angle (83.5° → 53°)); ↑ surface energy	PEEK-PDA-GO exhibited best wettability and uniform surface morphology	123
		CF-reinforced PEEK	GO–HAP: 0, 25, 50, 100 ng/mLGelMA/PEGDA	Freeze-drying	π–π and electrostatic bonding	↑ Surface porosity → ↑ NIR absorption → ↑ Photothermal conversion (33 °C→63 °C) → Stable cyclic heating → Enhanced hydrophilicity and cell adhesion	GH100 (100 ng/mL GO–HAP) → best photothermal & stability performance	205
	Convalent bonding	Epoxy resin	GO: 0.1 wt%-0.5 wt% HPEEK-g	Mechanical stirring + ultrasonication + curing	Covalent ester linkage (–COO–) between GO and HPEEK → improved interfacial adhesion and dispersion	↑ Storage modulus (+42 %) → ↑ hardness (+65 %) → ↑ fracture toughness (+31 %) → ↑ tensile strength (+7 %) → ↑ Tg (173–215 °C)	0.5 wt% HPEEK-g-GO → optimum mechanical and thermal enhancement	112
		Aminated PEEK	GO: 5–20 wt%	hot-press sintering	Covalent amide bonding (–NHCO–) between GO and PEEK → enhanced interfacial coupling and filler orientation	↑ Thermal conductivity (0.25 → 0.97 W/m·K, ↑380 %) → ↑ tensile stress (128 → 183 MPa) → ↑ Td (525.8 °C) → ↓ Tm/Tc (crystallinity suppression)	20 wt% GO → highest TC and Td; excessive GO reduces mechanical strength	113

#### 2.2.2 Surface coating techniques

Typical methods include dip-coating ([79], [80], [81], [82], [83], [84], [85], [86], [87], [88], [89], [90], [91], [92], [93]), vacuum-assisted filtration [108,109], self-assembly [[110], [111], [112]], and freeze-drying [54,113,114]. Non-covalent coatings are facile and minimally invasive to the substrate, enabling the introduction of antibacterial and osteogenic functions. Their limitations lie in weak interfacial adhesion and poor long-term stability, with delamination risks under physiological conditions. Covalent coatings form stable chemical bonds with the PEEK substrate, offering superior adhesion and long-term osseointegration potential. However, chemical modifications may compromise GO functional groups or trigger side reactions, necessitating strict control of processing parameters and biosafety. Recent studies on GO/HA/PEEK composite scaffolds have demonstrated in vitro mechanical and osteogenic properties comparable to cortical bone, underscoring their short-term feasibility [115] (Fig. 2) (Table 1).

In previous studies on GO-PEEK composites, the control of synthesis parameters has been identified as a critical determinant of their overall performance. Among these, GO concentration is the most frequently regulated factor. Most reports indicate that maintaining the GO content within 0.1–5 wt% markedly improves surface hydrophilicity, cellular adhesion, and osteogenic activity, whereas higher concentrations (>5 wt%) often result in agglomeration, interfacial defects, and excessive reactive oxygen species (ROS) generation, thereby compromising mechanical properties and increasing cytotoxicity [116]. The oxidation degree of GO is equally significant. A higher oxidation degree introduces abundant carboxyl, hydroxyl, and epoxy groups that facilitate protein adsorption and calcium deposition, but may simultaneously reduce conductivity and structural stability. In contrast, moderately oxidised GO has been shown to offer a more favorable balance between bioactivity and stability [117]. Surface exposure, largely determined by dispersion methods and fabrication processes, also plays a decisive role: uniformly dispersed and appropriately exposed GO nanosheets strongly promote cellular responses, whereas GO encapsulated within the PEEK matrix contributes little to biological performance [118]. Moreover, lateral size and layer thickness exert additional influences: small-flake or monolayer GO provides high surface area and uniform coating, while large-flake or multilayer GO is more effective for enhancing mechanical reinforcement [119]. Collectively, the interplay of GO concentration, oxidation degree, and surface exposure defines a “parameter window effect” that governs osteogenesis, antibacterial performance, and mechanical reinforcement. Optimising these parameters is therefore critical for advancing both fundamental research and clinical translation of GO-PEEK composites [120](Table 1).

In summary, melt extrusion or 3D printing combined with GO blending holds promise for load-bearing and structurally complex implants, whereas surface coating-particularly covalent modification-remains essential for enhancing bioactivity and osseointegration. Future development may favor hybrid approaches, such as integrating interfacial reinforcement with surface functionalization, to achieve synergistic optimization of mechanical robustness and biological performance.

## 3 Properties of GO-PEEK composites

### 3.1 Microstructure and surface characteristics

The incorporation of graphene oxide (GO) substantially modifies the surface morphology and interfacial properties of PEEK. GO nanosheets increase surface roughness and micro/nanostructures, which enhance protein adsorption, cell adhesion, and osteogenic differentiation [93,121]. Polar functional groups (–COOH, –OH) further promote hydrophilicity and Ca^2+^ deposition, thereby activating osteogenic signaling pathways. Within the bulk matrix, well-dispersed GO establishes strong interfacial bonding through π–π stacking, hydrogen bonding, or covalent grafting, facilitating stress transfer and improving composite stability. Conversely, GO aggregation may act as a stress concentrator and impair performance [57,58]. Emerging evidence indicates that GO can also modulate immune responses and promote angiogenesis, synergistically supporting osseointegration and long-term implant stability [122,123]. Thus, GO not only optimizes the physicochemical characteristics of PEEK but also endows it with biofunctional surfaces relevant for bone tissue engineering.

### 3.2 Mechanical properties

PEEK is widely used in orthopaedics due to its elastic modulus comparable to cortical bone; however, its bioinert surface remains a major barrier to osseointegration [35,36]. The introduction of GO as a filler enhances mechanical performance by improving interfacial adhesion and stress transfer via mechanical interlocking, π–π stacking, and hydrogen bonding [[57], [58], [59], [60], [61], [62],[65], [66], [67], [68], [69], [70], [71], [72], [73], [74],^76^,^77^,^81^]. Functionalized GO further amplifies these effects: amidated GO (AGO) improves hydrogen bonding with sulfonated PEEK (SPEEK), while phosphorylated GO (PGO), sulfonated GO (SGO), and epoxy-modified GO all demonstrate superior dispersion and interfacial stability [60,62,64,65]. Alternative strategies, such as polydopamine coating, in situ growth of metal–organic frameworks, and toluene-2,4-diisocyanate (TDI) functionalization, have achieved strong reinforcement and are promising for additive-manufactured PEEK components [58,61,72]. In addition, GO serves as an effective sizing agent for carbon nanotubes (CNTs) and carbon fibers (CFs), markedly improving stress transfer, toughness, and fatigue resistance [[66], [67], [68], [69], [70], [71],^77^]. While GO coatings exert minimal effects on bulk mechanical strength, they preserve robustness and significantly improve surface bioactivity [79,81,[85], [86], [87], [88], [89], [90],^93^,^111^,^124^]. Collectively, GO modification enables PEEK to maintain mechanical integrity while overcoming its intrinsic surface bioinertness(Table 1).

### 3.3 Photothermal effect and magnetic targeting properties

Beyond structural and mechanical advantages, GO imparts unique functional properties to PEEK composites. Its strong near-infrared (NIR) absorption and high photothermal conversion efficiency enable localized hyperthermia, with functionalized GO shown to inhibit melanoma growth under NIR irradiation [[125], [126], [127], [128], [129], [130], [131], [132], [133], [134]]. Integrating this capability into PEEK implants offers the potential for dual roles of structural support and local tumor ablation. Magnetic graphene oxide (MGO) further combines GO's physicochemical advantages with magnetic targeting. MGO has been demonstrated to promote osteogenic differentiation of bone marrow mesenchymal stem cells (BMSCs) under external magnetic fields while enabling controlled drug delivery [[134], [135], [136], [137], [138]]. MGO-based systems have shown synergistic chemo–photothermal efficacy in breast cancer and osteosarcoma models, and they hold promise as biosensors for detecting circulating tumor cells [[139], [140], [141], [142], [143], [144], [145], [146], [147], [148], [149], [150], [151]]. These multifunctional capabilities highlight GO-PEEK composites as next-generation implant materials that integrate mechanical compatibility with therapeutic and diagnostic functionalities, offering considerable translational potential in orthopaedic oncology and regenerative medicine.

## 4 Biological performance of GO-PEEK composites

### 4.1 Biocompatibility and cellular response

Biocompatibility is a fundamental prerequisite for bone repair materials, as it dictates the interactions between implants and host tissues and determines their physicochemical stability in vivo. An ideal orthopedic implant should minimize adverse host responses, particularly inflammation. [100,103,[152], [153], [154]]. Current evidence demonstrates that GO coatings exhibit negligible cytotoxicity toward osteoblasts and fibroblasts in vitro and only induce mild inflammatory reactions in vivo, underscoring their biosafety and clinical potential [79,80,104,155].

Through π–π stacking and hydrogen bonding, GO can firmly adhere to PEEK surfaces, significantly improving surface hydrophilicity and energy, thereby enhancing protein adsorption and promoting osteoblast/fibroblast adhesion and proliferation [88]. Multifunctional coatings that integrate GO with polydopamine, bioactive peptides, or inorganic components on sulfonated PEEK (SPEEK) have been reported to upregulate alkaline phosphatase (ALP) activity, calcium matrix deposition, and osteogenic gene expression [92]. Reduced GO (rGO) incorporated into 3D-printed PEEK scaffolds also shows promise for biomimetic bone repair [80], while GO combined with bioactive glass improves coating adhesion without compromising compatibility with osteoblasts and gingival fibroblasts [70]. In carbon fiber-reinforced PEEK (CFR-PEEK), GO modification enhances surface bioactivity and accelerates the early osteogenic response of bone marrow mesenchymal stem cells (BMSCs) [80]. Furthermore, GO-SPEEK has demonstrated the ability to induce hydroxyapatite (HAp) deposition and promote extracellular matrix mineralization in simulated body fluid [91].

Mechanistically, the biological activity of GO is largely attributed to its ability to improve hydrophilicity and provide functional groups (–COOH, –OH) that facilitate Ca^2+^ binding, mineral nucleation, and subsequent cell adhesion and osteogenic differentiation [122,123]. Recent findings further suggest that GO coatings may also enhance osseointegration via immunomodulation and pro-angiogenic effects, offering new strategies for complex bone defect repair [122].

### 4.2 Osteogenic activity and mineralization

**Fig. 3:**
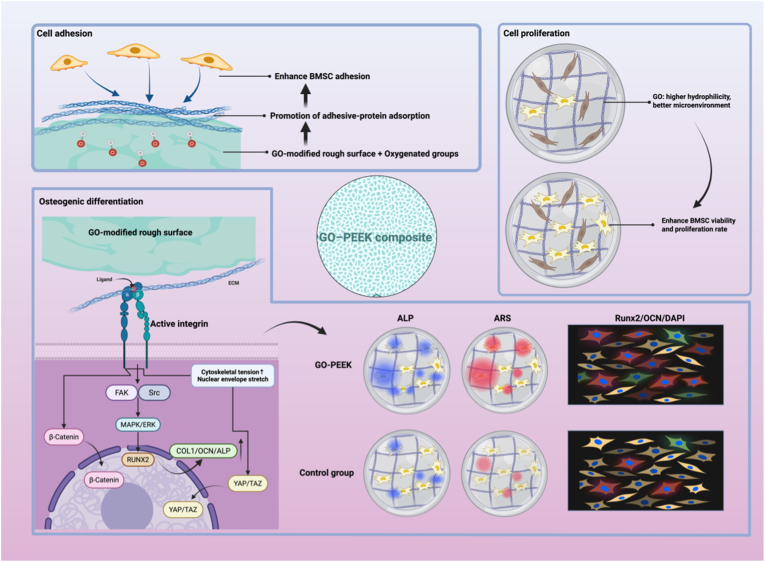
**Schematic illustration of the cellular responses to GO–PEEK composites**. GO-modified PEEK enhances BMSC adhesion through increased surface roughness and oxygen-containing functional groups, promoting adhesive protein adsorption. Improved hydrophilicity provides a favorable microenvironment for cell proliferation. The GO–PEEK surface further activates integrin-mediated signaling pathways (FAK/Src and MAPK/ERK), leading to upregulated expression of osteogenic markers (COL1, OCN, ALP, RUNX2, and YAP/TAZ) and enhanced osteogenic differentiation. Schematics created with BioRender.

In recent years, GO–PEEK composites have demonstrated unique potential in promoting osteogenic differentiation and mineral deposition. Their biological actions can be systematically interpreted from four interrelated perspectives: mechanotransduction, surface chemistry, osteoimmunomodulation, and angiogenesis, which collectively contribute to the “osteogenesis–mineralization” cascade (Fig. 3).

#### 4.2.1 Direct mechanisms (mechanotransduction)

The wrinkled morphology and nanoscale roughness of GO nanosheets provide multiple anchorage points and local tension gradients for cells, facilitating focal adhesion maturation (vinculin/FAK complex assembly) and activating the integrin–FAK–Src–MAPK cascade. This subsequently upregulates key osteogenic genes such as Runx2, COL1, and OCN, driving the “mechanosensing–signal transduction–osteogenic differentiation” process of MSCs [156,157](Fig. 3).

In parallel, the nanoscale topography of the substrate induces cytoskeletal reorganization and nuclear membrane stretching, enhancing β-catenin nuclear translocation and activating the Wnt/β-catenin pathway, which synergistically promotes osteogenic lineage commitment through multi-pathway crosstalk [158,159].

Moreover, graphene coatings significantly upregulate cell-surface integrins (e.g., α5β1), thereby activating the integrin/FAK axis and facilitating YAP/TAZ nuclear localization [156]. Previous studies in neuronal regeneration have also shown that GO stiffness and nanoscale roughness can co-enhance YAP/TAZ activation, which in osteogenesis promotes MSC proliferation and differentiation, cooperatively regulating Runx2 and ALP expression and subsequent mineral matrix deposition [160,161](Fig. 3).

#### 4.2.2 Indirect mechanisms (chemo–topography)

GO is inherently rich in polar functional groups such as –COOH and –OH, which markedly increase surface energy and hydrophilicity. These functional moieties preferentially adsorb fibronectin (FN) and vitronectin (VN), exposing RGD/LDV adhesion motifs and enhancing α5β1/αvβ3–integrin-mediated cell attachment and spreading, which in turn promotes proliferation and ALP expression [159,162,163].

In addition, the negatively charged GO surface can capture Ca^2+^ and PO_4_^3−^ ions, lowering the nucleation energy barrier for hydroxyapatite (HAp) formation and establishing a sequential process of ion enrichment → heterogeneous nucleation → mineral propagation. This cascade is accompanied by the amplification of BMP/Smad and integrin–FAK signaling, leading to ordered mineral deposition and matrix maturation [164,165](Fig. 3).

#### 4.2.3 Time- and concentration-dependent synergy

The osteogenic process of GO–PEEK composites follows a “initiation–accumulation–maturation” biological rhythm: At the early stage (1–7 days), oxygen-containing groups (–OH, –COOH) on GO enhance fibronectin adsorption by approximately 2.1-fold, facilitating MSC adhesion and proliferation while activating Runx2 and ALP. Consequently, ALP activity increases by ∼45 % compared with pure PEEK. During the intermediate phase (7–14 days), mineralization accelerates—Alizarin Red staining reveals a 60 % increase in mineral nodule formation compared with neat PEEK, with calcium and phosphate deposition reaching ∼1.2 mmol/L, comparable to native bone matrix levels. In the late phase (≥21 days), osseointegration progresses steadily; in vivo studies up to 12 weeks report a 35 % increase in bone–implant contact (BIC) ratio relative to unmodified PEEK, consistent with the long-term bone bridging behavior of PEEK fusion cages [71,75].

Concentration dependence exhibits a strict dose–window effect: The optimal GO loading lies between 0.05 and 0.1 wt%, at which the mineralized nodule area after 14 days increases by ∼25 % compared with 0.02 wt% without apparent cytotoxicity. Below 0.05 wt%, osteogenic signaling remains insufficient, while concentrations above 0.5 wt% generate excessive reactive oxygen species (ROS), impairing mineralization and reducing calcium deposition by ∼30 % after 21 days.

This concentration-dependent behavior parallels the trends observed in other polymeric GO composites, as discussed in the fabrication section(166, 167) (Table 1).

#### 4.2.4 Osteo-immune and angiogenic crosstalk

**Fig. 4:**
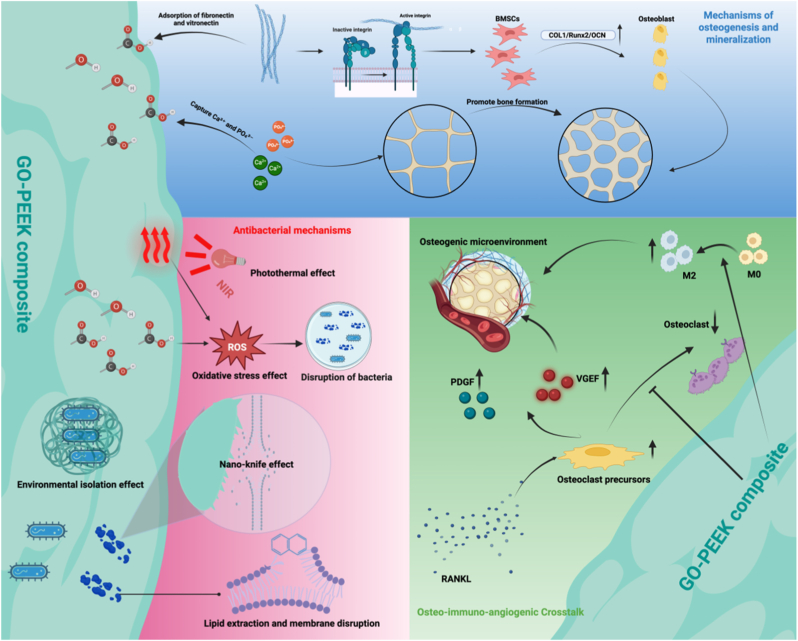
**Schematic illustration of the multifunctional biological mechanisms of GO–PEEK composites.** GO–PEEK composites promote osteogenesis by enhancing protein adsorption, activating integrin-mediated signaling, and stimulating osteogenic differentiation of BMSCs. The material also exhibits antibacterial effects through photothermal conversion, oxidative stress, and membrane disruption. Additionally, GO–PEEK regulates the osteo–immuno–angiogenic microenvironment by modulating macrophage polarization (M0→M2), osteoclast activity, and the release of VEGF, PDGF, and RANKL, thereby facilitating bone regeneration. Schematics created with BioRender.

GO also exerts its effects through immunomodulation and angiogenesis. It suppresses RANKL-induced osteoclast maturation and bone resorption, thereby restoring the osteoblast–osteoclast balance [168]. Simultaneously, GO promotes the release of platelet-derived growth factors and VEGF, enhancing angiogenic signaling, improving oxygen/nutrient supply, and facilitating recruitment of osteoprogenitor cells during early bone repair [122,169]. Moreover, GO surface chemistry and nano-topography promote macrophage polarization toward an M2 reparative phenotype, mitigating inflammation and favoring MSC osteogenic differentiation. These findings are consistent with reports that GO-SPEEK scaffolds enhance new bone formation in osteoporotic models [170](Fig. 4).

#### 4.2.5 Synergistic framework and design implications

Taken together, the osteogenic activity of GO-PEEK arises from a four-axis synergy: mechanotransduction, surface chemistry, osteo-immune regulation, and angiogenesis. Nano-topography initiates mechanosignaling, polar functional groups enhance protein/ion enrichment and nucleation, while immune regulation and angiogenesis reshape the local microenvironment. This integrated mechanism results in enhanced osteogenic differentiation, accelerated mineral deposition, and superior osseointegration [168].

Importantly, these effects follow a “window effect” determined by GO sheet size, surface density, and oxidation state. Suboptimal levels fail to establish stable protein coronas and ion-rich niches, whereas excessive levels may trigger ROS overproduction and cellular stress. Therefore, future designs should optimize GO within a safe–effective range, balancing osteogenic potency with biocompatibility. Particularly in challenging contexts such as osteoporosis and large-segment defects, establishing a “mineralization–immunity–angiogenesis” tri-axis equilibrium may represent a key strategy for clinical translation of GO-PEEK composites [166].

### 4.3 Antibacterial properties

PEEK and its derivatives (e.g., SPEEK) have emerged as promising candidates for orthopedic implants owing to their excellent mechanical strength, favorable biocompatibility, and mechanical similarity to native bone. However, their inherent lack of antibacterial activity remains a major clinical limitation, as implant-associated infections represent a leading cause of implant failure [171]. The incorporation of graphene oxide (GO) offers a potential strategy to overcome this challenge. GO-PEEK composites not only preserve the mechanical and biological advantages of PEEK but also confer multiple antibacterial mechanisms, exhibiting broad-spectrum inhibitory activity against both Gram-positive and Gram-negative bacteria [124].

#### 4.3.1 Primary antibacterial mechanisms

The antibacterial effects of GO can be summarized into five major categories.

(A) Physical disruption/Nano-knife effect: The sharp edges of GO nanosheets can penetrate bacterial membranes, leading to leakage of intracellular contents, irreversible damage, and eventual cell death [172].
(B) Oxidative stress: Oxygen-containing functional groups (–OH, –COOH, –O–) on GO promote the generation of reactive oxygen species (ROS). Excessive ROS induces oxidative damage to bacterial proteins, lipids, and DNA, resulting in metabolic dysfunction and death [173].
(C) Nutrient isolation: GO sheets can envelop bacterial membranes, disrupting nutrient transport and metabolic exchange, thereby suppressing bacterial viability [174].
(D) Lipid extraction and membrane disruption: The hydrophobic sp^2^ carbon framework of GO interacts with bacterial membranes, extracting phospholipids and compromising structural integrity [175].
(E) Photothermal/photodynamic effects: The π–π conjugated structure of GO enables broadband light absorption. Under near-infrared (NIR) irradiation, GO can simultaneously induce localized heating and ROS overproduction, achieving synergistic bactericidal effects [167,176].

#### 4.3.2 Mechanistic features in GO-PEEK composites

While these mechanisms remain largely applicable to GO-modified PEEK, their effectiveness depends on the degree of GO surface exposure and binding stability. Physical disruption and nutrient isolation effects may be attenuated due to immobilization of GO sheets within the matrix, yet strong interfacial adhesion minimizes the risk of nanosheet detachment and potential cytotoxicity. Consequently, antibacterial activity in GO-PEEK is predominantly maintained by direct contact-mediated killing and ROS-related pathways, which effectively inhibit bacterial adhesion and delay biofilm formation [75,91].

#### 4.3.3 Synergistic modifications and snhancement strategies

To further enhance antibacterial efficacy while maintaining biocompatibility, GO has been combined with various antibacterial agents:

Metallic/inorganic nanoparticles: GO/ZnO coatings display superior synergistic antibacterial activity compared with single components [177]; GO/nHA coatings improve both osseointegration and antibacterial effects [178]; GO/Ag-modified SPEEK surfaces achieve broad-spectrum antibacterial activity with lower cytotoxicity to osteogenic cells than Ag alone [179].

Bioactive molecules: SPEEK-GO-nisin coatings demonstrate potent antibiofilm formation and antibacterial properties [81]; GO combined with polydopamine (PDA) enhances antibacterial activity beyond that of either material alone [180].

#### 4.3.4 Design considerations and optimization

Although GO functionalization significantly augments the antibacterial performance of PEEK, balancing antibacterial efficacy with host cell compatibility remains a critical challenge. At high concentrations, GO may induce mild cytotoxicity; however, mammalian cells generally exhibit greater resilience and self-repair capacity, mitigating overall risks [181]. Future optimization should focus on: (A) precisely controlling GO concentration, lateral size, and oxidation state to avoid ROS-induced cytotoxicity; (B) refining deposition techniques to ensure stable adhesion and controlled exposure; and (C) developing multi-component hybrid systems to achieve dual antibacterial and osteogenic functionality(Fig. 4).

Summary,The antibacterial capability of GO-PEEK arises from the synergistic contributions of physical disruption, oxidative stress, nutrient isolation, membrane destabilization, and photothermal/photodynamic effects. Rational control of GO exposure, binding stability, and integration with complementary agents can suppress bacterial adhesion and biofilm formation while maintaining host cell safety, thereby advancing the clinical translation of GO-PEEK for orthopedic implants.

## 5 Applications of GO-PEEK composites in bone repair

### 5.1 In vitro studies

In vitro experiments are fundamental for evaluating the biological performance of GO-PEEK composites [182]. Common endpoints include cell adhesion, spreading, proliferation, and osteogenic differentiation. Studies have shown that GO-modified SPEEK significantly enhances adhesion and proliferation of osteoblasts and mesenchymal stem cells, while promoting calcium matrix deposition and upregulation of osteogenic genes [79]. When combined with dopamine, peptide molecules, or inorganic bioactive components, GO-modified SPEEK exhibits further improvements in ALP activity and mineralization [183]. In addition, reduced GO (rGO) integrated with 3D-printed PEEK scaffolds has demonstrated potential for biomimetic bone repair [72], while GO/bioactive glass composite coatings provide both strong interfacial bonding and excellent cytocompatibility [184]. Relevant studies have further confirmed that GO-SPEEK coatings significantly improve antibacterial activity and bioactivity while maintaining the intrinsic mechanical properties of the substrate [79]. GO-modified microporous SPEEK has also been shown to enhance osseointegration [185]. Nevertheless, several limitations remain. Precise control of GO concentration and surface exposure is challenging, and excessive levels may induce ROS-related cytotoxicity [186]. In addition, the long-term stability and degradation behavior of these coatings under complex physiological conditions are not yet fully understood [187]. Balancing antibacterial efficacy with host cell compatibility therefore represents a critical direction for further optimization [188] (Table 2).

**Table 2:** Summary of in vitro and in vivo bone-repair performance of GO–PEEK composites.

Sample type	Sample size	In vitro model	In vivo model	Main findings	Key results	Potential application	Ref.
GO-coated SPEEK	Ø 8.5 × 2 mm disks	Cell: MG63 Bacteria: *E. coli* and *S. aureus*	Not Performed	In vitro: GO–SPEEK exhibited improved hydrophilicity, enhanced MG-63 cell adhesion, proliferation, and osteogenic differentiation (↑ALP, RUNX2, COL1A1, OCN). Moderate antibacterial activity against *E. coli* (53.9 % at 0.5 wt%, 77.3 % at 1 wt%) compared with SPEEK.	0.5 wt% GO coating optimally enhances biocompatibility and antibacterial activity via improved surface wettability.	Orthopedic implants	18
GO-grafted CF reinforced PEEK	Ø 5 × 1 mm discs	Cells: Bone marrow mesenchymal stem cells (BMSCs) isolated from male Sprague Dawley(SD) rats	SD rats, bilateral calvarial defect (4–8 weeks)	In vitro: GO-CF/PEEK improved BMSC adhesion (4 × CF/PEEK) and proliferation. In vivo: No systemic toxicity; BV/TV, Tb.N ↑; Tb.Sp ↓; mineral deposition rate ↑2.2 × vs CF/PEEK.	GO–CF/PEEK exhibited excellent cytocompatibility promoted osteogenesis and new bone formation via enhanced surface bioactivity and BMSCs mechanotransduction	Orthopaedic/dental implants	66
GO-grafted CF reinforced PEEK	Ø 5 × 1 mm discs	Cells: BMSCs isolated from male SD rats	SD rats, bilateral calvarial defect (4–8 weeks)	In vitro: No cytotoxicity, enhanced BMSCs adhesion, proliferatin and osteogenic differentiation (↑Runx2, ALP, COl1a1, OCN) In vivo: No systemic toxicity, ↑BV/TV and ↑Tb.N, ↓Tb.Sp, and 2.2 × higher mineral deposition rate compared to CF/PEEK	AGO–CF/PEEK demonstrated excellent biocompatibility and promoted osteogenesis and new bone formation through enhanced surface bioactivity and BMSC responsiveness.	Orthopaedic/dental implants	69
GO-reinforced PEEK	Ø 8 × 1 mm discs	Cells: BMSCs isolated from SD rats	Not Performed	In vitro: BMSCs on 0.1–1.0 wt% GO/PEEK spread well with elongated pseudopodia; 0.5 wt% GO → best adhesion and proliferation; no cytotoxicity (CCK-8 ↑ over 1–7 days)	GO enhances BMSC adhesion and proliferation via improved hydrophilicity; 0.5 wt% GO = optimal for biocompatibility	Orthopaedic/dental implants	70
GO/HAp-reinforced PEEK	10 × 10 × 5 mm scaffolds	Cells: MG63	Male New Zealand white rabbits, radial diaphysis segmental bone defect (60 days)	In vitro: GO improved MG-63 cell adhesion, proliferation, and differentiation (↑ALP), ↑apatite formation In vivo: New bone bridged defect after 60 days; no systemic toxicity or inflammation.	GO acts as an interface phase to enhance interfacial bonding and bioactivity; promotes osteogenesis and bone regeneration via improved surface bioactivity	Bone tissue engineering scaffolds	71
GO-reinforced PVA/PEEK	Φ12 × 13 mm scaffolds	Cells: MG63	Male New Zealand rabbits, bone defect in middle of the radius diaphysis (8 weeks)	In vitro: Enhanced adhesion, proliferation, and ALP activity ↑ with time; ↑hydrophilicity and ↑degradation rate. In vivo: Radiographs and histology show complete defect bridging at 8 weeks, abundant new bone with ossein formation; no inflammation.	GO improves mechanical and biological performance; promotes osteogenesis and bone regeneration	Bone tissue engineering scaffolds	72
GO/BFP-SPEEK	Ø 8.5 × 2.5 mm (in vitro); Ø 4 × 10 mm (in vivo)	Cells: MC3T3-E1 Bacteria: *E. coli* and *S. aureus*	Rabbit, femoral defect (4–8 weeks)	In vitro: Enhanced MC3T3-E1 proliferation and osteogenic differentiation (↑ALP, RUNX2, OCN); >90 % antibacterial efficiency under NIR. In vivo: Accelerated osseointegration (↑BV/TV, ↑push-out strength), no inflammation	GO/pDA/BFP 2D coating synergistically promotes osteogenesis and photothermal antibacterial effect	Orthopedic implants	73
GO/BG-coated SPEEK	Not specified	Cells: MG63 and HGF-1 gingival fibroblasts Bacteria: *E. coli* and *S. aureus*	Not performed	In vitro: GO addition (0.75 wt%) enhanced antibacterial effect (larger inhibition zones, esp. vs *S. aureus*); no cytotoxicity to MG-63 or HGF-1, and induced apatite formation vs no GO group	BG/GO composite coating significantly improved hydrophilicity, adhesion, cytocompatibility, and antibacterial activity, suggesting promise for dental implant applications	Dental implants	74
GO/pDA-SPEEK	Ø 10 × 2 mm discs	Cells: MC3T3-E1 Bacteria: *E. coli* and *S. aureus*	Not performed	In vitro: GO–SPEEK exhibited highest antibacterial rate (*E. coli* ↓ 86 %, *S. aureus* ↓ 94 %), superior MC3T3-E1 adhesion and spreading with elongated filopodia, significantly increased cell viability, and upregulated osteogenic genes (↑RUNX2, OCN, COL-I)	GO coating synergizes with sulfonation to markedly enhance surface hydrophilicity and bioactivity, enabling strong osteogenic differentiation and antibacterial activity without affecting mechanical stability	Orthopaedic/dental implants	75
GO/ZnO-coated SPEEK	10 × 10 × 2 mm discs	Cells: L929; Bacteria: *S. sanguinis*, *F. nucleatum* and *P. gingivalis*	Not performed	In vitro: Cell response: No cytotoxicity observed, L929 cells adhered and spread well. Antibacterial activity: inhibition rates of *S. sanguinis* ≈ 97 %, *P. gingivalis* ≈ 89 %, *F. nucleatum* ≈ 39 %. Significantly reduced CFU counts and suppressed biofilm formation;	ZnO/GO-SPEEK coating provided effective antibacterial activity against peri-implant pathogens while maintaining excellent cytocompatibility; potential for dental implant abutment applications	Orthopaedic implants	76
rGO-coated PEEK	20 × 10 × 5 mm porous scaffolds	Not specificed	Not performed	In vitro: rGO–PEEK exhibited enhanced cell adhesion, proliferation, and viability over 14 days	rGO–PEEK scaffolds showed improved surface morphology, cytocompatibility, and mechanical strength; 3 wt% rGO identified as optimal for bone implant applications	Bone implants	77
GO/nHA-coated PEEK	Ø 16 mm discs	: MG63 and GF-1 fibroblasts Bacteria: *E. coli* and *S. aureus*	Not performed	In vitro: Cell response: HGF viability (75.1 %) > MG-63 (68.2 %) Antibacterial: *S. aureus*↓ ≈ 99 % and *E. coli* (>2 log reduction); slight antibacterial effect of nHA alone	nHA/GO coating significantly improved antibacterial performance and maintained acceptable cytocompatibility, supporting potential as a bioactive, antibacterial surface for PEEK-based dental implants.	Dental implants	80
GO/nisin-PEEK/SPEEK	Not specified	*S. aureus*	Not performed	In vitro: PEEK/SPEEK → no inhibition → dense biofilm → GO → inhibition zone 10 mm → nisin → 19 mm → GO–nisin → 27 mm → no bacterial adhesion or biofilm → severe membrane rupture under SEM	GO + nisin = synergistic antibacterial & anti-adhesion → dual mechanism: ROS (GO) + membrane disruption (nisin)	Orthopedic/dental implants	81
GO/MFO-PEEK/SPEEK	Not specified	Bacteria: *E. coli* and *S. aureus*	Not performed	In vitro: Without NIR → partial killing via Fe^3+^/Mn^3+^ release and GSH oxidation → with NIR (808 nm, 10 min) → 99.99 % bactericidal rate → visible bacterial rupture (SEM)	Combined GPx-mimetic & photothermal effects cause ROS imbalance and hyperthermia → synergistic antibacterial mechanism	Bone implants	82
GO-coated CF-PEEK	10 × 10 × 1 mm (in vitro), Ø5 mm × 1 mm (in vivo)	Cells: BMSCs and L929	SD rats, calvarial defect, (1–3 months)	In vitro: GO increased cell attachment, proliferation ( × 3 vs CF/PEEK), ↑ALP activity (4.19 ± 0.08 U/mg protein vs 1.70 ± 0.07), ↑mineralization In vivo: ↑bone ingrowth; no cytotoxicity (RGR >75 %), no systemic toxicity in heart/liver/kidney	GO wrapping promoted osteogenic differentiation, mineralization, and osseointegration while maintaining biosafety and mechanical stability	Orthopedic/dental implants	83
GO/HAp-coated CFR–SPEEK	10 × 10 × 2 mm discs	Cells: MC3T3–E1	Not performed	In vitro: ↑ Cell viability on all coatings (p < 0.0001); HAp-coated CFR–SPEEK → highest proliferation at 120 h (vs 10 %RGO); ↑ Filopodia extensions and adhesion on all coated surfaces	Sulfonation + HAp/rGO coating enhanced surface wettability and biocompatibility; HAp-only coating showed best osteogenic cell response	Bone-contact implant surfaces/orthopedic fixation	87
GO/FDA-PEEK/SPEEK	Not specified	Cells: RAW264.7 macrophages and BMSCs(co-culture)	OVX rat, femoral defect	In vitro: ↓ STAT3–NLRP3/caspase-1/IL-1β signaling → ↓ inflammation; ↑ IL-4, IL-10 → anti-inflammatory; ↓ M1 (CCR7^+^ 51.6 %→18.0 %) ↑ M2 (CD206^+^ 17.6 %→47.4 %); ↑ALP, OCN, RUNX2 expression; ↓ osteoclast TRAP^+^ cells → less bone resorption; In vivo: ↑ BV/TV, Tb.Th, BMD → enhanced osseointegration	SPEEK@PDA–GO inhibited osteoclastogenesis, promoted M2 macrophage polarization and osteogenesis via STAT3/NLRP3 axis suppression	Osteoporotic implants	90
GO-SPEEK	10 × 10 × 1 mm discs	Cells: MC3T3-E1; Bacteria: P. gingivalis and S. mutans	Not performed	In vitro: Cell response: ↑ adhesion and proliferation, and osteogenesis: ↑ALP, ↑ARS mineralisation ↑ Antibacterial: P. gingivalis ↓ 80.8 %, S. mutans ↓ 66.4 % vs PEEK	GO coating on 3D SPEEK enhances hydrophilicity, antibacterial activity and osteogenic differentiation without affecting mechanical properties; promising for orthopaedic/dental implants.	Orthopaedic/dental implants	91
GO/Ag–SPEEK	Not specified	Cells: MC3T3-E1; Bacteria: *E. coli*, *S. aureus*, *P. aeruginosa*, *K. pneumoniae* and *C. albicans*	Not performed	In vitro: Antibacterial: GO–SPEEK → ↓ CFU (10 %) → limited effect; Ag/GO–SPEEK → ↓ CFU (*E. coli* ↓25 %, *S. aureus* ↓22 %) → clear inhibition zones; ↓ biofilm formation (OD_575_ ↓38.6 %) → disrupted bacterial membranes → Ag^+^ sustained release (0.36 μg/mL @ 3 days) Cell response: biocompatible with osteoblasts	Ag/GO double decoration → dual antibacterial mechanism (Ag^+^ release + GO anti-adhesion) → suppressed biofilm formation → low cytotoxicity	Bone implants	92
GO-coated CF-SPEEK	10 × 10 × 3 mm (in vitro)/Ø 5 mm discs (in vivo)	Cells: L929 and BMSCs	SD rats, skull defect, (4–12 weeks)	In vitro: Cell reponse: Cell viability >75 %; ↑Osteogenic markers (↑ALP, Runx-2, Col-I, OCN ↑ > 2-fold vs PEEK); In vivo: Micro-CT showed abundant new bone and improved osseointegration; no visceral toxicity	Sulfonation + GO coating synergistically improved hydrophilicity, apatite formation, osteogenic gene expression and osseointegration, while maintaining mechanical strength and biosafety	Orthopedic/dental implants	94
GO-coated PEEK	1 × 1 cm^2^ discs	Cells: C2C12 murine cell lines	Not performed	In vitro: ↓ Cytotoxicity (<6 % LDH release) → high cell viability; ↑ Cell adhesion & spreading → enhanced myogenic differentiation; plasma-GO > dip-GO in stability	GO coatings showed excellent cytocompatibility and enhanced adhesion; plasma treatment improved stability and cell response	Muscle or bone interface materials	95
GO/PDA-coated PEEK	Not specified	Cells: human gingival fibroblasts (HGFs) Bacteria: *P. gingivalis*, *F. nucleatum*, and *S. mutans*.	Not performed	In vitro: ↓ CFU counts (PAG < PA < P); ↑ ROS-mediated bacterial membrane rupture; ↓ virulence gene expression (Fim, Gtf, FadA ↓); ↑ fibroblast adhesion & viability (>95 %)	PDA–GO (PAG) surface achieved strong antibacterial activity via oxidative stress and membrane disruption while maintaining cytocompatibility	Dental implants	123
GO/HAp-reincorced PEEK	12.4 mm × 12.4 mm × 6 mm scaffolds	Cells: NIH3T3 mouse embryonic fibroblast	Not Performed	In vitro: GO/HAp-PEEK (5 wt% GO, 30 wt% HAp) scaffolds exhibited highest cell viability compared to GO-PEEK scaffold, 30 wt% HAp promote cells adhesion and proliferation, while 40 wt% slightly inhibits	5 wt% GO significantly improves PEEK-HAp interfacial bonding and homogeneity; 30 % HA provides an optimal balance between mechanical and cellular activity	Bone tissue engineering scaffolds	104
GO/HAp reinforced PEEK	10 mm × 10 mm × 1 mm scaffolds	Not specificed	Not Performed	In vitro: Biological activity and osteogenesis are significantly improved	Addition of rGO + cHAp improved PEEK's bioactivity and osteogenesis; rGO/cHAp well-dispersed in PEEK matrix, enhancing cytocompatibility	Orthopaedic implants	155
GO/PDA@ZrO-PEEK	Not specified	Cells: L929 and BMSCs Bacteria: *E. coli* and *S. aureus*	Rabbit, cranial defect model (4–12 weeks)	In vitro: BMSC adhesion & proliferation → ↑ ALP & ARS under NIR → antibacterial rate: 99.4 % (*E. coli*), 95.8 % (*S. aureus*) → no hemolysis In vivo: new bone fills lock grooves → Mortise-and-tenon integration after 8 wk	PGPZ (+NIR) exhibited superior photothermal-enhanced osteogenesis and osseointegration without toxicity or inflammation	Bone implants	167
GO/HAp-PEEK	Not specified	Cells: BMSCs and Human Umbilical Vein Endothelial Cells(HUVECs) Bacteria: *E. coli* and *S. aureus*	Not performed	In vitro: Without NIR → partial antibacterial effect (via GO–bacteria contact) → With NIR → T↑ to 63 °C → bacterial membrane damage & protein denaturation → antibacterial rate 97.79 % (*S. aureus*), 96.09 % (*E. coli*) → excellent BMSC & HUVEC viability (no cytotoxicity)	GO–HAP cryogel modification provided synergistic photothermal and biocompatibility enhancement	Bone implant	206

Future in vitro assessments should emphasize [1]: direct comparisons with reference materials such as pristine PEEK and titanium [2]; short-term (hours to 3 days) and long-term (7–21 days or more) dynamic observations [3]; multi-endpoint evaluations, including protein adsorption, osteogenic gene/protein expression, and mineralization assays; and [4] the influence of GO content on bioactivity and potential cytotoxicity. Overall, in vitro studies not only verify the advantages of GO-PEEK but also provide mechanistic insights and guidance for compositional optimization.

### 5.2 In vivo studies

Building upon in vitro evidence, GO-PEEK composites have been tested in various animal bone defect models, including calvarial, femoral, and tibial defects, to evaluate osteogenesis and osseointegration [189]. Key parameters include bone–implant contact (BIC), bone volume fraction (BV/TV), bone mineral density (BMD), micro-CT analysis, push-out/pull-out mechanical testing, and histological assessment. Results consistently indicate that GO-modified SPEEK or CFR-PEEK exhibits superior bone integration and new bone formation compared with controls [190,191]. Notably, GO-PEEK composites have also been associated with reduced inflammatory responses and enhanced angiogenesis in vivo, suggesting potential utility for complex bone defect repair [72] (Table 2).

Critical considerations from animal studies include [1]: strong and stable binding of GO to the PEEK surface to avoid delamination [2]; precise control of GO content and distribution for long-term stability; and [3] balancing immunomodulatory and osteogenic effects. These findings provide essential preclinical evidence supporting the clinical translation of GO-PEEK.

### 5.3 Clinical translation potential

At present, no GO-PEEK implants have entered human clinical trials [192]; however, their translational prospects have attracted increasing attention. Based on current preclinical evidence, the clinical advantages of GO-PEEK are mainly reflected in three aspects.

(i) an elastic modulus more closely matched to bone tissue, which may reduce stress shielding and improve long-term stability [193,194];
(ii) significant enhancement of antibacterial and osteogenic performance by GO modification in vitro and in vivo, potentially reducing infection risk and promoting osseointegration [195,196];
(iii) multifunctional properties—including antibacterial, osteoinductive, and immunomodulatory effects—that provide integrated therapeutic strategies for complex defects and high-infection-risk scenarios [90].

Recent studies have also demonstrated that pristine PEEK implants have been successfully applied in maxillofacial and neurosurgical defect repair with favorable preliminary outcomes, thereby offering a material and engineering foundation for GO-based composites [197]. In addition, systematic reviews suggest that GO-based biomaterials possess unique advantages and versatility in bone repair [198].

#### 5.3.1 Post-tumor (drug-loading + osteogenesis) reconstruction

Segmental defects following bone tumor resection require both structural reconstruction and prevention of local recurrence and infection [199]. GO nanosheets, rich in carboxyl and hydroxyl groups, can act as carriers for chemotherapeutics, antibacterial agents, and osteogenic factors, thus enabling a dual function of “localized release + bone regeneration” [200]. Compared with systemic delivery, local administration achieves higher drug concentrations at the lesion site with reduced systemic toxicity, while concurrently suppressing bacterial adhesion and biofilm formation [201]. Moreover, the photothermal/photodynamic (PTT/PDT) properties of GO under NIR irradiation enable tumor cell ablation and synergize with drug or antibacterial release, constituting a “therapy–repair integration” strategy that combines oncological control with bone regeneration [202].

Potential anti-tumor applications of GO-PEEK include.

**Fig. 5:**
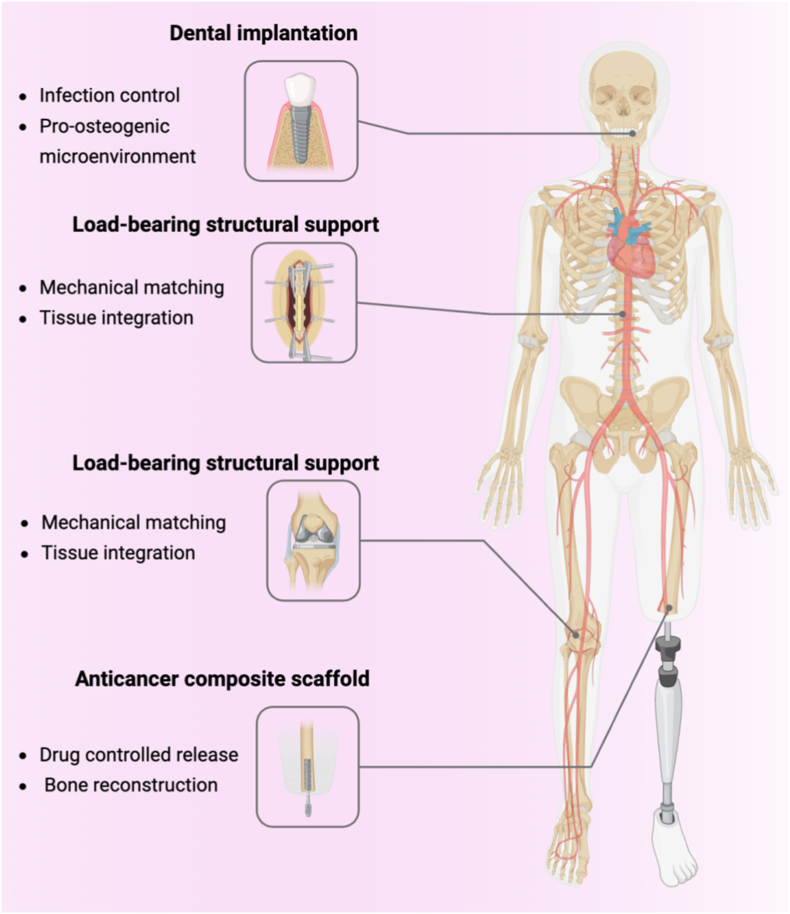
**Representative biomedical applications of GO–PEEK composites.** GO–PEEK composites are applied in dental implantation for infection control and promotion of a pro-osteogenic microenvironment; in load-bearing implants such as spinal, joint, and trauma supports for mechanical matching and tissue integration; and in anticancer scaffold for controlled drug release and bone reconstruction. Schematics created with BioRender.

(a) Localized chemotherapy delivery (LC-Tx): GO can load small-molecule chemotherapeutics (e.g., anthracyclines, taxanes) or targeted inhibitors, offering tunable release kinetics and reduced systemic toxicity. Co-delivery of antibacterial agents may further minimize infection risk [203].
(b) Photothermal/photodynamic synergy (PTT/PDT): GO's broadband absorption and ROS generation capacity allow NIR-triggered local ablation of residual tumor cells, with potential synergism when combined with chemotherapy or antibacterial release [167,204,205].
(c) Immune microenvironment modulation: GO's surface chemistry and topography can influence macrophage polarization and inflammatory cascades. Coupled with immunoregulatory molecules, this approach may optimize “inflammation control–bone regeneration,” improving postoperative outcomes [206].
(d) Osteogenesis and structural repair: GO-PEEK has demonstrated improved osteogenic signaling and bone–implant integration in vitro and animal models, facilitating defect healing and biomechanical restoration [132].
(e) Imaging and follow-up: Leveraging the radiolucency and MRI compatibility of PEEK, GO-PEEK implants reduce artifacts and enhance accuracy in postoperative imaging, thereby supporting long-term monitoring and recurrence surveillance [207](Fig. 5).

#### 5.3.2 Dental implants and alveolar bone repair

In dentistry, infection remains one of the primary causes of implant failure. Both GO-SPEEK coatings and GO-modified microporous SPEEK exhibit strong antibacterial activity and osteogenic potential in vitro, effectively reducing bacterial adhesion and biofilm formation while promoting osteoblast adhesion, proliferation, and differentiation [208]. These features render GO-PEEK particularly suitable for low-load sites such as alveolar bone repair, where it can reduce postoperative infection risk and accelerate osseointegration. Considering the ease of follow-up and replacement of dental implants, the alveolar bone represents a promising early entry point for the clinical translation of GO-PEEK(Fig. 5).

#### 5.3.3 Joint implants and spinal fusion devices

PEEK has been extensively utilized in spinal fusion devices and various internal fixation implants across orthopaedic subspecialties, including joint and trauma surgery [33]. Its outstanding radiolucency under X-ray and MRI, favorable mechanical compatibility with bone tissue, and excellent machinability have made it one of the preferred materials for orthopaedic load-bearing applications [209]. However, its bioinertness and susceptibility to infection remain limitations. GO modification has the potential to mitigate bacterial adhesion, reduce infection risk, and enhance long-term fusion stability [210]. Nevertheless, given the complex biomechanical environment, further studies are required to assess the long-term stability of GO coatings, interfacial bonding strength, and safety with respect to wear debris. A rational translational pathway would involve initial application in small-scale fusion devices or lower-risk cases, followed by stepwise expansion to large-scale, load-bearing applications(Fig. 5).

## 6 Challenges and future directions

### 6.1 Material-level challenges

The long-term stability and degradation behavior of graphene oxide (GO) remain key barriers to clinical translation. In physiological and immune environments, GO is readily degraded by reactive oxygen species secreted by neutrophils and macrophages, leading to compromised coating integrity and particle-related risks [211,212]. Furthermore, inappropriate GO concentration or oxidation state may provoke excessive ROS production, resulting in cytotoxicity or chronic inflammation [213]. Future work should focus on establishing a structure–property–safety paradigm that systematically correlates GO sheet size, oxidation degree, and functional group exposure with stability and biocompatibility. In parallel, the integration of GO with biodegradable polymers or ceramics may enable controlled degradation of functional layers, maintaining mechanical support while mitigating long-term risks [214,215].

### 6.2 Functional-level challenges

The multifunctionality of GO raises significant concerns regarding the balance of biological effects. Antibacterial activity and osteogenic stimulation can be antagonistic: potent antibacterial actions may suppress osteoblast viability, whereas osteogenic promotion may attenuate antibacterial efficacy [216,217]. Moreover, the photothermal and photodynamic (PTT/PDT) effects of GO rely on near-infrared (NIR) irradiation, yet the limited penetration depth and substantial attenuation in deep bone or peri-metallic regions restrict their clinical feasibility [218]. Addressing these issues requires temporally staged multifunctional strategies, in which antibacterial efficacy is prioritized in the immediate postoperative phase, followed by the sequential release of osteogenic and immunomodulatory cues to achieve dynamic synergy [[219], [220], [221]]. Additional refinements may be achieved through surface functionalization and bioactive ligand conjugation, enabling selective modulation of bacterial, osteogenic, and immune cellular responses [222].

### 6.3 Translational-level challenges

The industrialization and clinical translation of GO-PEEK remain hindered by systemic challenges. Batch-to-batch consistency is difficult to ensure, as variations in GO sheet size, oxidation state, and surface exposure lead to unpredictable biological outcomes [216]. Process standardization is insufficient, with a paucity of GMP-compliant protocols and traceable quality systems capable of meeting regulatory requirements [217,223]. Sterilization compatibility remains unresolved: conventional methods (γ-irradiation, ethylene oxide, steam sterilization) may alter GO chemistry, undermining antibacterial and osteogenic performance [224]. In addition, the increased manufacturing complexity and cost associated with GO functionalization raise questions of economic viability, which must be justified by demonstrable clinical benefits such as reduced infection, accelerated healing, and fewer revision surgeries. Such benefits require validation in prospective clinical trials and real-world studies [169].

Future translational strategies should prioritize: (i) defining a design space that links GO concentration, exposure, and clinical outcomes within quality-by-design frameworks [120]; (ii) developing 3D printing–compatible, patient-specific processes that ensure coating uniformity and long-term stability in complex geometries [225]; and (iii) implementing a stepwise clinical pathway, beginning with low-load, high-monitorability indications (e.g., dental implants, cranial defects) and progressively expanding to load-bearing long bone prostheses and spinal/joint/tumor implantss, supported by cost-effectiveness analyses [226].

## 7 Conclusions and outlook

GO-PEEK has emerged as a next-generation orthopaedic biomaterial that retains the mechanical and imaging advantages of PEEK while imparting enhanced antibacterial, osteogenic, and immunomodulatory properties [91]. GO-SPEEK coatings and GO-modified microporous SPEEK have demonstrated promising outcomes in dental implantation, post-tumor reconstruction, and spinal/joint implants, highlighting their translational potential [79].

Nonetheless, three critical hurdles remain: (i) material-level concerns regarding long-term stability and particle-associated risks [227,228]; (ii) functional-level challenges in reconciling antibacterial, osteogenic, and immune-regulatory effects, along with the clinical feasibility of NIR-based stimulation [229]; and (iii) translational-level barriers including batch consistency, process standardization, sterilization compatibility, and cost-effectiveness validation [230,231].

Looking ahead, progress should be driven along three interconnected axes—materials, functionality, and translation. Key priorities include.

(A) the establishment of structure–property–safety models and rigorous multiscale in vivo validation [232];
(B) the implementation of temporally staged, multifunctional release strategies to harmonize antibacterial, immunomodulatory, and osteogenic effects [233];
(C) the development of GMP-compliant manufacturing workflows and regulatory-ready evidence chains supported by real-world data [234]; and
(D) the integration of GO-PEEK with 3D printing, digital modeling, and intelligent drug-delivery systems to enable personalized, smart implants [235].

In conclusion, GO-PEEK presents a compelling clinical value proposition characterized by strong material advantages, synergistic functional enhancements, and well-defined application scenarios. With robust advances in safety profiling, process standardization, and evidence-based translation, GO-PEEK is well positioned to advance from laboratory validation to clinical reality, offering a multifunctional solution for infection-prone and complex bone repair.

## Declaration of Generative AI in scientific writing

During the preparation of this work the author(s) used ChatGPT-4 in order to improve readability and refine the language. After using this tool/service, the author(s) reviewed and edited the content as needed and take(s) full responsibility for the content of the publication.

## Authors’ contributions

MJZ and SZL conceived the review topic and wrote the manuscript. JYX, MJZ, and NT prepared and drew the figures. AS, LQNg, CNS and XS contributed to the literature collection, data organization, and interpretation. SZL and CZL provided critical revision for important intellectual content and technical accuracy. All authors have read and approved the final manuscript

## Funding

The work was funded by the first batch of Young Scholars Support Programme of Peking Union Medical College (project no: 2022002) & EU via HORIZON - MSCA-2024-DN- RENOVATE project (project no: 101227121). The funders have no role in the study design, data collection and analysis, decision to publish or preparation of the manuscript.

## Declaration of competing interest

The authors declare that they have no known competing financial interests or personal relationships that could have appeared to influence the work reported in this paper.

## Abbreviations

**GO**: Graphene oxide
**AGO**: Amidated graphene oxide
**PGO**: Phosphorylated graphene oxide
**SGO**: Sulfonated graphene oxide
**PEEK**: Poly(Ether Ether keton)
**SPEEK**: Sulfonated Poly(Ether Ether Ketone)
**APEEK**: Aminated PEEK
**HPEEK**: Hydroxylated PEEK
**DMSO**: Dimethyl Sulfoxide
**DMF**: N,N-Dimethylformamide
**DMAc**: N,N-Dimethylacetamide
**DCC**: Dicyclohexylcarbodiimide
**DMAP**: 4-Dimethylaminopyridine
**CF**: Carbon fibre
**PDA**: Polydopamine
**BFP**: Bone-forming oligopeptide
**pDA**: Polydopamine
**nHA**: Nano hydroxypatite
**AgNPs**: Silver nano particles
**MFO**: MnFe_2_O_4_
**PTT**: Photothermal therapy
**PDT**: Photodynamic therapy

## References

[1] Shi W.P., Jiang Y.P., Wu T.Y., Zhang Y.Z., Li T. (2024). Advancements in drug-loaded hydrogel systems for bone defect repair. Regen Ther.

[2] Zhang B., Yin X.H., Zhang F., Hong Y.R., Qiu Y.S., Yang X.Y. (2023). Customized bioceramic scaffolds and metal meshes for challenging large-size mandibular bone defect regeneration and repair. Regen Biomater.

[3] Han Y.F., Wu Y., Wang F.X., Li G.F., Wang J., Wu X. (2024). Heterogeneous DNA hydrogel loaded with Apt02 modified tetrahedral framework nucleic acid accelerated critical-size bone defect repair. Bioact Mater.

[4] Habibovic P. (2017). (∗) strategic directions in osteoinduction and biomimetics. Tissue Eng Part A.

[5] Bharadwaz A., Jayasuriya A.C. (2020). Recent trends in the application of widely used natural and synthetic polymer nanocomposites in bone tissue regeneration. Mater Sci Eng C Mater Biol Appl.

[6] He P., Zhao Y.B., Wang B., Liu G.Y., Zhang L., Li M. (2024). A biodegradable magnesium phosphate cement incorporating chitosan and rhBMP-2 designed for bone defect repair. J Orthop Transl.

[7] Liao S.N., Li S.H., Liu Z.Q., Lu W.T., He Y.T., Xia K. (2024). A bioswitchable siRNA delivery system: RNAi therapy based on tetrahedral framework nucleic acids for bone defect repair. Nanoscale.

[8] Yu D., Tang Z., Bao S., Guo S., Chen C., Wu Q. (2025). Immunoregulatory neuro-vascularized osseointegration driven by different nano-morphological CaTiO(3) bioactive coatings on porous titanium alloy scaffolds. Adv Healthc Mater.

[9] Ghorbani F., Ghalandari B., Detsch R., Liu C., Boccaccini A.R. (2025). TGF-β1/BSA coating modulates multi-phasic scaffolds for osteochondral tissue regeneration. Mater Today Bio.

[10] Tian Y.Z., Sun R.L., Li Y.F., Liu P., Fan B., Xue Y. (2024). Research progress on the application of magnesium phosphate bone cement in bone defect repair: a review. Bio Med Mater Eng.

[11] Zhou J.C., See C.W., Sreenivasamurthy S., Zhu D.H. (2023). Customized additive manufacturing in bone scaffolds-the gateway to precise bone defect treatment. Research.

[12] Gelli R., Ridi F. (2023). An overview of magnesium-phosphate-based cements as bone repair materials. J Func Biomater.

[13] Zou M., Sun J.C., Xiang Z. (2021). Induction of M2-Type macrophage differentiation for bone defect repair via an interpenetration network hydrogel with a GO-Based controlled release system. Adv Healthc Mater.

[14] Acs Applied Nano Materialswang H, Zhang J.X., Bai H.T., Wang C.Y., Li Z.H., Wang Z.H. (2025). 3D printed cell-free bilayer porous scaffold based on alginate with biomimetic microenvironment for osteochondral defect repair. Biomater Adv.

[15] Wei F.L., Zhai Y., Wang T.F., Zhao J.W., Wang C.L., Tang Z. (2024). Stem cell-homing biomimetic hydrogel promotes the repair of osteoporotic bone defects through osteogenic and angiogenic coupling. Sci Adv.

[16] Zou M., Sun J., Xiang Z. (2020). [Early effect of graphene oxide-carboxymethyl chitosan hydrogel loaded with interleukin 4 and bone morphogenetic protein 2 on bone immunity and repair]. Zhongguo Xiu Fu Chong Jian Wai Ke Za Zhi.

[17] Li H., Guan Z., Wei L., Lu J., Tan Y., Wei Q. (2024). In situ co-deposition synthesis for collagen-Astragalus polysaccharide composite with intrafibrillar mineralization as potential biomimetic-bone repair materials. Regen Biomater.

[18] Ma H.Y., Suonan A.X., Zhou J.Y., Yuan Q.L., Liu L., Zhao X.M. (2021). PEEK (Polyether-ether-ketone) and its composite materials in orthopedic implantation. Arab J Chem.

[19] Chen T.J., Jinno Y., Atsuta I., Tsuchiya A., Stocchero M., Bressan E. (2023). Current surface modification strategies to improve the binding efficiency of emerging biomaterial polyetheretherketone (PEEK) with bone and soft tissue: a literature review. J Prosthodont Res.

[20] Lin L.Y., Pei X.Q., Bennewitz R., Schlarb A.K. (2018). Friction and wear of PEEK in continuous sliding and unidirectional scratch tests. Tribol Int.

[21] de Ruiter L., Rankin K., Browne M., Briscoe A., Janssen D., Verdonschot N. (2021). Decreased stress shielding with a PEEK femoral total knee prosthesis measured in validated computational models. J Biomech.

[22] Joerger A.K., Seitz S., Lange N., Aftahy A.K., Wagner A., Ryang Y.M. (2022). CFR-PEEK pedicle screw instrumentation for spinal neoplasms: a single center experience on safety and efficacy. Cancers.

[23] El-Hajje A., Kolos E.C., Wang J.K., Maleksaeedi S., He Z.M., Wiria F.E. (2014). Physical and mechanical characterisation of 3D-printed porous titanium for biomedical applications. J Mater Sci Mater Med.

[24] Huang H., Liu X., Wang J., Suo M., Zhang J., Sun T. (2024). Strategies to improve the performance of polyetheretherketone (PEEK) as orthopedic implants: from surface modification to addition of bioactive materials. J Mat Chem B.

[25] Clunk M.J., Gonzalez M.R., Denwood H.M., Werenski J.O., Sodhi A., Hoffman B.A. (2023). A PEEK into carbon fiber: a practical guide for high performance composite polymeric implants for orthopaedic oncology. J Orthop.

[26] Di Maggio B., Sessa P., Mantelli P., Maniscalco P., Rivera F., Calori G.M. (2017). PEEK radiolucent plate for distal radius fractures: Multicentre clinical results at 12 months follow up. Injury-Int J Care Inj.

[27] Thippeswamy P.B., Nedunchelian M., Rajasekaran R.B., Riley D., Khatkar H., Rajasekaran S. (2021). Updates in postoperative imaging modalities following musculoskeletal surgery. J Clin Orthop Trauma.

[28] Ge Y.M., Hu L.Q., Liu J.Y., Ma F.B., Zhang J.R., Wang Y.S. (2024). Peek@ZIF-8(CEL) as a novel bone implant for large defect repair and enhanced bone healing via a long-term stable bioactive releaser. ACS Appl Mater Interfaces.

[29] Xu L., Li M.X., Ma F.B., Zhang H.G., Liang X.J., Cheng G.Y. (2024). Surface bioactivation of polyetheretherketone (PEEK) by magnesium chondroitin sulfate (MgCS) as orthopedic implants for reconstruction of skeletal defects. Int J Biol Macromol.

[30] Smith J.A., Basgul C., Mohammadlou B.S., Allen M., Kurtz S.M. (2024). Investigating the feasibility and performance of hybrid overmolded UHMWPE 3D-Printed PEEK structural composites for orthopedic implant applications: a pilot study. Bioengineering-Basel.

[31] Xu X.D., Wang L., Wang J.M., Yu X.C., Huang W.M. (2024). Retrieval analysis of PEEK rods pedicle screw system: three cases analysis. BMC Musculoskelet Disord.

[32] Wu H., Guo Y., Guo W. (2023). Effect of carbon-fiber-reinforced polyetheretherketone on stress distribution in a redesigned tumor-type knee prosthesis: a finite element analysis. Front Bioeng Biotechnol.

[33] Cheers G.M., Weimer L.P., Neuerburg C., Arnholdt J., Gilbert F., Thorwächter C. (2024). Advances in implants and bone graft types for lumbar spinal fusion surgery. Biomater Sci.

[34] Ahmad F., Nimonkar S., Belkhode V., Nimonkar P. (2024). Role of polyetheretherketone in prosthodontics: a literature review. Cureus.

[35] Zheng W.Z., Wu D.X., Zhang Y.W., Luo Y.K., Yang L., Xu X.R. (2023). Multifunctional modifications of polyetheretherketone implants for bone repair: a comprehensive review. Biomater Adv.

[36] Panayotov I.V., Orti V., Cuisinier F., Yachouh J. (2016). Polyetheretherketone (PEEK) for medical applications. J Mater Sci Mater Med.

[37] Li M.J., Liu J.Y., Li Y.T., Chen W.Y., Yang Z., Zou Y.Y. (2024). Enhanced osteogenesis and antibacterial activity of dual-functional PEEK implants via biomimetic polydopamine modification with chondroitin sulfate and levofloxacin. J Biomater Sci Polym Ed.

[38] Zhang J.J., Ma T.T., Liu X.Y., Zhang X.R., Meng W.Q., Wu J.L. (2024). Multifunctional surface of the nano-morphic PEEK implant with enhanced angiogenic, osteogenic and antibacterial properties. Regen Biomater.

[39] Yang H., Ding H.Y., Tian Y., Wu C., Chen Y.B., Shi H.X. (2024). Metal element-fusion peptide heterostructured nanocoatings endow polyetheretherketone implants with robust anti-bacterial activities and in vivo osseointegration. Nanoscale.

[40] Wu Y.Z., Huo S.C., Liu S., Hong Q.M., Wang Y., Lyu Z. (2023). Cu-Sr bilayer bioactive glass nanoparticles/polydopamine functionalized polyetheretherketone enhances osteogenic activity and prevents implant-associated infections through spatiotemporal immunomodulation. Adv Healthc Mater.

[41] Chen Y.H., Chen Y.Y., Han T.L., Xie Z., Yang Y.C., Chen S.Y. (2023). Enhanced osteogenic and antibacterial properties of polyetheretherketone by ultraviolet-initiated grafting polymerization of a gelatin methacryloyl/epsilon-poly-L-lysine/laponite hydrogel coating. J Biomed Mater Res Part A.

[42] Bobyn J.D., Stackpool G.J., Hacking S.A., Tanzer M., Krygier J.J. (1999). Characteristics of bone ingrowth and interface mechanics of a new porous tantalum biomaterial. J Bone Joint Surg-Br.

[43] Deporter D.A., Watson P.A., Pilliar R.M., Melcher A.H., Winslow J., Howley T.P. (1986). A histological assessment of the initial healing response adjacent to porous-surfaced, titanium alloy dental implants in dogs. J Dent Res.

[44] Grizon F., Aguado E., Huré G., Baslé M.F., Chappard D. (2002). Enhanced bone integration of implants with increased surface roughness:: a long term study in the sheep. J Dent.

[45] Sun C., Zhao H., Wang L., Zhang J., Zheng J., Yang Z. (2022). Additive manufactured polyether-ether-ketone composite scaffolds with hydroxyapatite filler and porous structure promoted the integration with soft tissue. Biomater Adv.

[46] Abdallah M.N., Badran Z., Ciobanu O., Hamdan N., Tamimi F. (2017). Strategies for optimizing the soft tissue seal around osseointegrated implants. Adv Healthc Mater.

[47] Wang L., Yang C.C., Sun C.N., Yan X.L., He J.K., Shi C.Q. (2022). Fused deposition modeling PEEK implants for personalized surgical application: from clinical need to biofabrication. Int J Bioprinting.

[48] Hu X.J., Huang J.C., Wei Y.Z., Zhao H.Y., Lin S.Z., Hu C.X. (2022). Laser direct-write sensors on carbon-fiber-reinforced poly-ether-ether-ketone for smart orthopedic implants. Adv Sci.

[49] De Salvatore S., Longo U.G., Vincenzi B., Pantano F., Zollo G., Calabrese G. (2024). Clinical performance of implanted devices used in surgical treatment of patients with spinal tumors: a systematic review. BMC Musculoskelet Disord.

[50] Castro J.I., Payan-Valero A., Valencia-Llano C.H., Zapata M.E.V., Hernández J.H.M., Zapata P.A. (2024). Graphene oxide nanosheets for bone tissue regeneration. Molecules.

[51] Kashte S.B., Kadam S., Maffulli N., Potty A.G., Migliorini F., Gupta A. (2024). Osteoinductive potential of graphene and graphene oxide for bone tissue engineering: a comparative study. J Orthop Surg Res.

[52] Cheng Y., Dong H., Wu Y.Y., Xiao K.J. (2021). Preparation of an amidated graphene oxide/sulfonated poly ether ether ketone (AGO/SPEEK) modified atmosphere packaging for the storage of cherry tomatoes. Foods.

[53] Cai Y.Y., Zhang Q.G., Zhu A.M., Liu Q.L. (2021). Two-dimensional metal-organic framework-graphene oxide hybrid nanocomposite proton exchange membranes with enhanced proton conduction. J Colloid Interface Sci.

[54] Ye J.Y., Wu C., Qin W., Zhong F.F., Ding M. (2020). Advanced sulfonated Poly(Ether Ether Ketone)/Graphene-Oxide/Titanium dioxide nanoparticle composited membrane with superior cyclability for vanadium redox flow battery. J Nanosci Nanotechnol.

[55] Das A.K., Manohar M., Shahi V.K. (2018). Cation-exchange membrane with low frictional coefficient and high limiting current density for energy-efficient water desalination. ACS Omega.

[56] Liu Y.R., Wu W.J., Li P., Lin J.L., Yang Z.H., Wang J. (2019). Constructing long-range transfer pathways with ordered acid base pairs for highly enhanced proton conduction. ACS Appl Mater Interfaces.

[57] Cao N., Zhou C.F., Wang Y., Ju H., Tan D.Y., Li J. (2018). Synthesis and characterization of sulfonated graphene oxide reinforced sulfonated poly (Ether Ether Ketone) (SPEEK) composites for Proton exchange membrane materials. Materials.

[58] Qiu X., Ueda M., Hu H.Y., Sui Y.G., Zhang X., Wang L.J. (2017). Poly(2,5-benzimidazole)-Grafted graphene oxide as an effective proton conductor for construction of nanocomposite proton exchange membrane. ACS Appl Mater Interfaces.

[59] Jiang Z.J., Jiang Z.Q., Tian X.N., Luo L.J., Liu M.L. (2017). Sulfonated Holey Graphene Oxide (SHGO) filled sulfonated poly(ether ether ketone) membrane: the role of holes in the SHGO in improving its performance as proton exchange membrane for direct methanol fuel cells. ACS Appl Mater Interfaces.

[60] Wu W.J., Li Y.F., Chen P.P., Liu J.D., Wang J.T., Zhang H.Q. (2016). Constructing ionic liquid-filled proton transfer channels within nanocomposite membrane by using functionalized graphene oxide. ACS Appl Mater Interfaces.

[61] Zhang N., Wang B.L., Zhang Y.R., Bu F.Z., Cui Y., Li X.F. (2014). Mechanically reinforced phosphoric acid doped quaternized poly(ether ether ketone) membranes via cross-linking with functionalized graphene oxide. Chem Commun.

[62] Hu C.X., Liu T.H., Neate N., Fay M., Hou X.H., Grant D. (2022). Enhanced thermal and electrical properties by Ag nanoparticles decorated GO-CNT nanostructures in PEEK composites. Compos Sci Technol.

[63] Hwang Y., Kim M., Kim J. (2013). Improvement of the mechanical properties and thermal conductivity of poly(ether-ether-ketone) with the addition of graphene oxide-carbon nanotube hybrid fillers. Compos Pt A-Appl Sci Manuf.

[64] Song H.J., Li N., Li Y.J., Min C.Y., Wang Z. (2012). Preparation and tribological properties of graphene/poly(ether ether ketone) nanocomposites. J Mater Sci.

[65] Ren T.N., Zhu G.M., Hou X., Li B., Hao Y.J. (2021). Improvement of interfacial interactions in CF/PEEK composites by an s-PSF/graphene oxide compound sizing agent. J Appl Polym Sci.

[66] Qin W., Xing T., Qin S.N., Tang B., Chen W.Y. (2024). BMSCs-driven graphite oxide-grafted-carbon fibers reinforced polyetheretherketone composites as functional implants: in vivo biosafety and osteogenesis. J Biomater Sci Polym Ed.

[67] Yang C., Xu J., Xing Y., Hao S.J., Ren Z.D. (2020). Covalent polymer functionalized graphene oxide/poly(ether ether ketone) composites for fused deposition modeling: improved mechanical and tribological performance. RSC Adv.

[68] Chen C., Meng L.H., Hu Y.R., Su Z.N., Zhang T.T., Ouyang Z.Y. (2021). Graphene oxide-reinforced poly (ether-ether-ketone)/silica composites with improved mechanical performance and surface bioactivity. J Mech Behav Biomed Mater.

[69] Qin W., Xing T., Tang B., Chen W.Y. (2023). Mechanical properties and osteogenesis of CFR-PEEK composite with interface strengthening by graphene oxide for implant application. J Mech Behav Biomed Mater.

[70] He M.M., Chen X.C., Guo Z.J., Qiu X.T., Yang Y.T., Su C.L. (2019). Super tough graphene oxide reinforced polyetheretherketone for potential hard tissue repair applications. Compos Sci Technol.

[71] Peng S.P., Feng P., Wu P., Huang W., Yang Y.W., Guo W. (2017). Graphene oxide as an interface phase between polyetheretherketone and hydroxyapatite for tissue engineering scaffolds. Sci Rep.

[72] Feng P., Jia J.Y., Peng S.P., Yang W.J., Bin S.Z., Shuai C.J. (2020). Graphene oxide-driven interfacial coupling in laser 3D printed PEEK/PVA scaffolds for bone regeneration. Virtual Phys Prototyp.

[73] Wang S., Duan C.Y., Yang W.Z., Gao X.Y., Shi J.C., Kang J.P. (2020). Two-dimensional nanocoating-enabled orthopedic implants for bimodal therapeutic applications. Nanoscale.

[74] Al-Noaman A., Rawlinson S.C.F. (2023). A novel bioactive glass/graphene oxide composite coating for a polyether ether ketone-based dental implant. Eur J Oral Sci.

[75] Huang R., Gu Y.J., Yuan Y.J., Wang Y.X., Pan Y.S., Li B. (2024). A self-assembling graphene oxide coating for enhanced bactericidal and osteogenic properties of poly-ether-ether-ketone. Front Bioeng Biotechnol.

[76] Yang S.H., Yu W.Q., Zhang J.J., Han X., Wang J.Y., Sun D. (2022). The antibacterial property of zinc oxide/graphene oxide modified porous polyetheretherketone against S. sanguinis, F. nucleatum and P. gingivalis. Biomed Mater.

[77] Oladapo B.I., Ismail S.O., Ikumapayi O.M., Karagiannidis P.G. (2022). Impact of rGO-coated PEEK and lattice on bone implant. Colloids Surf B Biointerfaces.

[78] Kang E.S., Kim H., Han Y., Cho Y.W., Son H., Luo Z. (2021). Enhancing osteogenesis of adipose-derived mesenchymal stem cells using gold nanostructure/peptide-nanopatterned graphene oxide. Colloids Surf B Biointerfaces.

[79] Ouyang L., Deng Y., Yang L., Shi X.Y., Dong T.S., Tai Y.Y. (2018). Graphene-Oxide-Decorated Microporous Polyetheretherketone with Superior Antibacterial Capability and In Vitro Osteogenesis for Orthopedic Implant. Macromol Biosci.

[80] Al-Noaman A., Rawlinson S.C.F. (2023). Polyether ether ketone coated with nanohydroxyapatite/graphene oxide composite promotes bioactivity and antibacterial activity at the surface of the material. Eur J Oral Sci.

[81] Kumar S.R., Hu C.C., Vi T.T.T., Chen D.W., Lue S.J. (2023). Antimicrobial peptide conjugated on graphene oxide-containing sulfonated polyetheretherketone substrate for effective antibacterial activities against Staphylococcus aureus. Antibiotics (Basel).

[82] Yang H., Wu C., Deng Y., Yang W.Z., He M.M., Zhang L. (2024). MnFe2O4/graphene oxide modified PEEK with phototherapeutic and GPx-mimetic potential for anti-bacterial treatment. Mater Lett.

[83] Qin W., Li Y., Ma J., Liang Q., Cui X.H., Jia H. (2020). Osseointegration and biosafety of graphene oxide wrapped porous CF/PEEK composites as implantable materials: The role of surface structure and chemistry. Dent Mater.

[84] Oladapo B.I., Zahedi S.A. (2021). Improving bioactivity and strength of PEEK composite polymer for bone application. Mater Chem Phys.

[85] Baligidad S.M., Arunkumar T., Thodda G., Elangovan K. (2023). Fabrication of HAp/rGO nanocomposite coating on PEEK: tribological performance study. Surf Interfaces.

[86] Coa F., Delite F.D., Strauss M., Martinez D.S.T. (2022). Toxicity mitigation and biodistribution of albumin corona coated graphene oxide and carbon nanotubes in Caenorhabditis elegans. NanoImpact.

[87] Asante N.A., Wang Y.F., Bakhet S., Kareem S., Owusu K.A., Hu Y.D. (2021). Ambient temperature sulfonated carbon fiber reinforced PEEK with hydroxyapatite and reduced graphene oxide hydroxyapatite composite coating. J Biomed Mater Res Part B.

[88] Lin Z.Y., Zhong J.D., Sun R.Y., Wei Y.Z., Sun Z.H., Li W.Y. (2023). InSitu integrated fabrication for multi-interface stabilized and highly durable polyaniline@graphene oxide/polyether ether ketone special separation membranes. Adv Sci.

[89] Zhang W.P., Zhang J., Bao T., Zhou W., Meng J.W., Chen Z.L. (2013). Universal multilayer assemblies of graphene in chemically resistant microtubes for microextraction. Anal Chem.

[90] Yang C., Zhu K.C., Cheng M.Q., Yuan X.W., Wang S.J., Zhang L. (2024). Graphene oxide-decorated microporous sulfonated polyetheretherketone for guiding osteoporotic bone regeneration. J Control Release.

[91] Guo C., Lu R., Wang X., Chen S. (2021). Antibacterial activity, bio-compatibility and osteogenic differentiation of graphene oxide coating on 3D-network poly-ether-ether-ketone for orthopaedic implants. J Mater Sci Mater Med.

[92] Wei W., Zhu J., Liu Y., Chen L., Zhu W., Ji H. (2024). Graphene oxide-silver-coated sulfonated polyetheretherketone (Ag/GO-SPEEK): a broad-spectrum antibacterial artificial bone implants. ACS Appl Bio Mater.

[93] Peng C., Iqbal Z., Sirkar K.K., Peterson G.W. (2020). Graphene oxide-based membrane as a protective barrier against toxic vapors and gases. ACS Appl Mater Interfaces.

[94] Li Y., Jia H., Cui X., Qin W., Qin S., Wu Y. (2022). Bending properties, compression properties, biocompatibility and bioactivity of sulfonated carbon Fibers/PEEK composites with graphene oxide coating. Appl Surf Sci.

[95] Awaja F., Tripathi M., Coraça-Huber D., Speranza G. (2018). Biocompatibility of different graphene oxide coatings on polymers. Materialia.

[96] Hung H.S., Shen C.C., Wu J.T., Yueh C.Y., Yang M.Y., Yang Y.C. (2024). Assessment of the biocompatibility ability and differentiation capacity of mesenchymal stem cells on biopolymer/gold nanocomposites. Int J Mol Sci.

[97] Castro J.I., Valencia Llano C.H., Tenorio D.L., Saavedra M., Zapata P., Navia-Porras D.P. (2022). Biocompatibility assessment of polylactic acid (PLA) and nanobioglass (n-BG) nanocomposites for biomedical applications. Molecules.

[98] Sydlik S.A., Jhunjhunwala S., Webber M.J., Anderson D.G., Langer R. (2015). In vivo compatibility of graphene oxide with differing oxidation states. ACS Nano.

[99] Di Crescenzo A., Zara S., Di Nisio C., Ettorre V., Ventrella A., Zavan B. (2019). Graphene oxide foils as an osteoinductive stem cell substrate. ACS Appl Bio Mater.

[100] Baligidad S.M., Chethan Kumar G., Maharudresh A.C., Lekshmi I.C., Rajasree S., Pillai R. (2022). Investigation on strain rate sensitivity of 3D printed sPEEK-HAP/rGO composites. J Manuf Process.

[101] Sankar S., Paulraj J., Chakraborti P., Jeyaseelan C. (2025). Development of graphene oxide and hydroxyapatite reinforced PEEK composites: a bio-tribological study with different stroke length and loading conditions. J Polym Res.

[102] Zhang H., Li Y., Jiang N., Zhou N., Zou X., Zhang D. (2025). Synergistic modified poly (ether ether ketone) with the transition layer constructed by graphene oxide grafted macromolecular. Surf Interfaces.

[103] Sathishkumar S., Paulraj J., Chakraborti P., Chandradass J., Ghosh S.K. (2024). Mechanical and tribological assessment of PEEK and PEEK based polymer composites for artificial hip joints. Int J Mater Res.

[104] Karthic M., Chockalingam K., Vignesh C., Nagarajan K.J. (2024). Enhancement of mechanical and biological properties of PEEK/GO/HA composite scaffolds fabricated through 3D printing and sintered process for bone tissue engineering. Polym Plast Tech Mater.

[105] Wang Y., Xia W., Giuntoli A. (2025). Optimizing graphene dispersion via polymer grafting. Macromolecules.

[106] Li S., Jia C., Han H., Yang Y., Xiaowen Y., Chen Z. (2024). Characterization and biocompatibility of a bilayer PEEK-based scaffold for guiding bone regeneration. BMC Oral Health.

[107] Dallal S., Eslami B., Tiari S. (2025). Recent advances in PEEK for biomedical applications: a comprehensive review of material properties, processing, and additive manufacturing. Polymers.

[108] He M.M., Zhu C., Sun D., Liu Z., Du M.X., Huang Y. (2022). Layer-by-layer assembled black phosphorus/chitosan composite coating for multi-functional PEEK bone scaffold. Compos Pt B Eng.

[109] Saravanan S., Chawla A., Vairamani M., Sastry T.P., Subramanian K.S., Selvamurugan N. (2017). Scaffolds containing chitosan, gelatin and graphene oxide for bone tissue regeneration in vitro and in vivo. Int J Biol Macromol.

[110] Katti P., Kundan K.V., Kumar S., Bose S. (2018). Poly(ether ether ketone)-Grafted graphene oxide "Interconnects" enhance mechanical, dynamic mechanical, and flame-retardant properties in epoxy laminates. ACS Omega.

[111] Katti P., Kundan K.V., Kumar S., Bose S. (2017). Improved mechanical properties through engineering the interface by poly (ether ether ketone) grafted graphene oxide in epoxy based nanocomposites. Polymer.

[112] Zheng Y.Y., Xiong C.D., Zhang S.L., Li X.Y., Zhang L.F. (2015). Bone-like apatite coating on functionalized poly(etheretherketone) surface via tailored silanization layers technique. Mater Sci Eng C-Mater Biol Appl.

[113] Sun Y.L., Dang H.B., Li X.N., Luan J.S., Jiang D., Mu J.X. (2024). Enhanced thermal conductivity of PEEK based composites fabricated by its fibers grafted graphene oxide. Colloid Surf A-Physicochem Eng Asp.

[114] Jia M.S., Hash S., Reynoso W., Elsaadany M., Ibrahim H. (2023). Characterization and biocompatibility assessment of boron nitride magnesium nanocomposites for orthopedic applications. Bioengineering-Basel.

[115] Mi L., Li F., Xu D., Liu J., Li J., Zhong L. (2024). Performance of 3D printed porous polyetheretherketone composite scaffolds combined with nano-hydroxyapatite/carbon fiber in bone tissue engineering: a biological evaluation. Front Bioeng Biotechnol.

[116] Hou Y.H., Wang W.G., Bartolo P. (2024). The effect of graphene and graphene oxide induced reactive oxygen species on polycaprolactone scaffolds for bone cancer applications. Mater Today Bio.

[117] Cámara-Torres M., Sinha R., Eqtesadi S., Wendelbo R., Scatto M., Scopece P. (2021). Effect of the reduced graphene oxide (rGO) compaction degree and concentration on rGO-polymer composite printability and cell interactions. Nanoscale.

[118] Kumari S., Singh D., Srivastava P., Singh B.N., Mishra A. (2022). Generation of graphene oxide and nano-bioglass based scaffold for bone tissue regeneration. Biomed Mater.

[119] Chen Y.L., Zhu Z.Y., Shen Y., Liu X.L., He Y.S., Lyu C. (2025). Evaluating the potential of graphene oxide to promote skeletal muscle complex regeneration. Front Bioeng Biotechnol.

[120] Gu Y., Miao F.Y., Liu K.J., Su Y.M., Wei Y., Hu Y.C. (2023). Fabrication of gelatin methacryloyl/graphene oxide conductive hydrogel for bone repair. J Biomater Sci Polym Ed.

[121] Marshall K.M., Wojciechowski J.P., Jayawarna V., Hasan A., Echalier C., Ovrebo O. (2024). Considerations of growth factor and material use in bone tissue engineering using biodegradable scaffolds in vitro and in vivo. Sci Rep.

[122] Liu W.J., Wang Q.Y., Luo H.Y., Luo B.C., Zhao F.J., Kang Y.Y. (2024). Nanographene oxide promotes angiogenesis by regulating osteoclast differentiation and platelet-derived growth factor secretion. ACS Nano.

[123] Hiew V.V., Teoh P.L. (2024). Differential gene expression of Wharton's jelly-derived mesenchymal cells mediated by graphene oxide in basal and osteo-induced media. Mol Biol Rep.

[124] Guo C., Lu R., Wang X., Chen S. (2021). Graphene oxide-modified polyetheretherketone with excellent antibacterial properties and biocompatibility for implant abutment. Macromol Res.

[125] Zhou X., Jiang J., Dang J., Wang Y., Hu R., Shen C. (2024). Intelligent supramolecular modification for implants: endogenous regulation of bone defect repair in osteoporosis. Adv Mater.

[126] Chen M., Qiao Y., Yu L., Wang W., Wang W., Sun H. (2025). A microenvironment responsive polyetheretherketone implant with antibacterial and osteoimmunomodulatory properties facilitates osseointegration. Bioact Mater.

[127] Hu W., Peng C., Luo W., Lv M., Li X., Li D. (2010). Graphene-based antibacterial paper. ACS Nano.

[128] Gurunathan S., Han J.W., Dayem A.A., Eppakayala V., Kim J.H. (2012). Oxidative stress-mediated antibacterial activity of graphene oxide and reduced graphene oxide in Pseudomonas aeruginosa. Int J Nanomedicine.

[129] Hu C., Yang Y., Lin Y., Wang L., Ma R., Zhang Y. (2021). GO-based antibacterial composites: application and design strategies. Adv Drug Deliv Rev.

[130] Rojas-Andrade M.D., Chata G., Rouholiman D., Liu J., Saltikov C., Chen S. (2017). Antibacterial mechanisms of graphene-based composite nanomaterials. Nanoscale.

[131] Wang Y., Li J., Li X., Shi J., Jiang Z., Zhang C.Y. (2022). Graphene-based nanomaterials for cancer therapy and anti-infections. Bioact Mater.

[132] Tang Z., Zhao L., Yang Z., Liu Z., Gu J., Bai B. (2018). Mechanisms of oxidative stress, apoptosis, and autophagy involved in graphene oxide nanomaterial anti-osteosarcoma effect. Int J Nanomedicine.

[133] Chen X., Sun Z., Peng X., Meng N., Ma L., Fu J. (2024). Graphene oxide/black phosphorus functionalized collagen scaffolds with enhanced near-infrared controlled in situ biomineralization for promoting infectious bone defect repair through PI3K/Akt pathway. ACS Appl Mater Interfaces.

[134] Liu D., Fu J., Fan H.B., Li D.C., Dong E.C., Xiao X. (2018). Application of 3D-printed PEEK scapula prosthesis in the treatment of scapular benign fibrous histiocytoma: a case report. J Bone Oncol.

[135] Wang L., Huang L.J., Li X.F., Zhong D.X., Li D.C., Cao T.S. (2019). Three-dimensional printing PEEK implant: a novel choice for the reconstruction of chest wall defect. Ann Thorac Surg.

[136] Boriani S., Pipola V., Cecchinato R., Ghermandi R., Tedesco G., Fiore M.R. (2020). Composite PEEK/carbon fiber rods in the treatment for bone tumors of the cervical spine: a case series. Eur Spine J.

[137] Bhashyam A.R., Yeung C., Sodhi A., Xu R.F., Groot O.Q., Kelly S. (2023). Titanium vs. carbon fiber-reinforced intramedullary nailing for humeral bone tumors. J Shoulder Elb Surg.

[138] Guo Y., Chen C., Zhang S., Ren L., Zhao Y., Guo W. (2022). Mediation of mechanically adapted TiCu/TiCuN/CFR-PEEK implants in vascular regeneration to promote bone repair in vitro and in vivo. J Orthop Translat.

[139] Cofano F., Di Perna G., Monticelli M., Marengo N., Ajello M., Mammi M. (2020). Carbon fiber reinforced vs titanium implants for fixation in spinal metastases: a comparative clinical study about safety and effectiveness of the new "carbon-strategy". J Clin Neurosci.

[140] Baez D.F. (2023). Graphene-based nanomaterials for photothermal therapy in cancer treatment. Pharmaceutics.

[141] Babakhani A., Peighambardoust S.J., Olad A. (2024). Fabrication of magnetic nanocomposite scaffolds based on polyvinyl alcohol-chitosan containing hydroxyapatite and clay modified with graphene oxide: evaluation of their properties for bone tissue engineering applications. J Mech Behav Biomed Mater.

[142] Chen H.Y., Fan Y.Z., Shi Z.F., Liu C.L., Ran M.F., Zhai J.X. (2024). NIR-responsive micropatterned nanocomposite functionalized implant for sequential antibacterial and osteogenesis. Colloid Surf B-Biointerfaces.

[143] Wu K.Y., Chen Y.X., Zhang Q.Q., Gu Y., Liu R., Luo J. (2024). Preparation of graphene oxide/polymer hybrid microcapsules via photopolymerization for double self-healing anticorrosion coatings. ACS Appl Mater Interfaces.

[144] Guncum E., Geyik G., Isiklan N. (2024). Magnetic graphene oxide functionalized alginate-g-poly (2-hydroxypropylmethacrylamide) nanoplatform for near-infrared light/pH/magnetic field-sensitive drug release and chemo/phototherapy. Int J Pharm.

[145] Yin D., Li Y., Lin H., Guo B., Du Y., Li X. (2013). Functional graphene oxide as a plasmid-based Stat3 siRNA carrier inhibits mouse malignant melanoma growth in vivo. Nanotechnology.

[146] Kalluru P., Vankayala R., Chiang C.S., Hwang K.C. (2016). Nano-graphene oxide-mediated in vivo fluorescence imaging and bimodal photodynamic and photothermal destruction of tumors. Biomaterials.

[147] Zhan X.Z., Teng W.Q., Sun K., He J.X., Yang J., Tian J.H. (2021). CD47-mediated DTIC-loaded chitosan oligosaccharide-grafted nGO for synergistic chemo-photothermal therapy against malignant melanoma. Mater Sci Eng C-Mater Biol Appl.

[148] Nettore B.P.V., Prarnanik A., Chavva S.R., Sinha S.S., Robinson C., Fan Z. (2014). Aptamer-conjugated theranostic hybrid graphene oxide with highly selective biosensing and combined therapy capability. Faraday Discuss.

[149] Shlapakova L.E., Pryadko A.S., Zharkova I.I., Volkov A., Kozadaeva M., Chernozem R.V. (2024). Osteogenic potential and long-term enzymatic biodegradation of PHB-based scaffolds with composite magnetic nanofillers in a magnetic field. ACS Appl Mater Interfaces.

[150] Sarikhani A.R., Abedi M., Abolmaali S.S., Borandeh S., Tamaddon A.M. (2024). Magnetic graphene oxide nanosheets with amidoamine dendronized crosslinks for dual pH and redox-sensitive doxorubicin delivery. BMC Chem.

[151] He Y., Chen G.H., Li Y., Li Y.M., Yi C., Zhang X.L. (2021). Effect of magnetic graphene oxide on cellular behaviors and osteogenesis under a moderate static magnetic field. Nanomed Nanotechnol Biol Med.

[152] Habibovic P., Barralet J.E. (2011). Bioinorganics and biomaterials: bone repair. Acta Biomater.

[153] Wei C.B., Liu Z.F., Jiang F.F., Zeng B.H., Huang M.D., Yu D.S. (2017). Cellular behaviours of bone marrow-derived mesenchymal stem cells towards pristine graphene oxide nanosheets. Cell Prolif.

[154] Sankar S., Chakraborti P., Jeyaseelan C., Paulraj J. (2025). Tribological analysis on hybrid PEEK-based polymer composites for prosthetic implant applications. J Tribol.

[155] Oladapo B.I., Zahedi S.A., Ismail S.O. (2021). Mechanical performances of hip implant design and fabrication with PEEK composite. Polymer.

[156] Xie H., Cao T., Franco-Obregón A., Rosa V. (2019). Graphene-induced osteogenic differentiation is mediated by the Integrin/FAK axis. Int J Mol Sci.

[157] Wu M.S., Zou L., Jiang L.L., Zhao Z.H., Liu J. (2021). Osteoinductive and antimicrobial mechanisms of graphene-based materials for enhancing bone tissue engineering. J Tissue Eng Regen Med.

[158] Soleymani H., Moghaddam M.M., Naderi-Manesh H., Taheri R.A. (2024). Single-layer graphene oxide nanosheets induce proliferation and Osteogenesis of single-cell hBMSCs encapsulated in Alginate microgels. Sci Rep.

[159] Shim N.Y., Heo J.S. (2021). Performance of the polydopamine-graphene oxide composite substrate in the osteogenic differentiation of mouse embryonic stem cells. Int J Mol Sci.

[160] Catanesit M., Panella G., Benedetti E., Fioravanti G., Perrozzi F., Ottaviano L. (2018). YAP/TAZ mechano-transduction as the underlying mechanism of neuronal differentiation induced by reduced graphene oxide. Nanomedicine.

[161] Tang Y., Weiss S.J. (2017). Snail/Slug-YAP/TAZ complexes cooperatively regulate mesenchymal stem cell function and bone formation. Cell Cycle.

[162] Zhang Y., Nayak T.R., Hong H., Cai W.B. (2012). Graphene: a versatile nanoplatform for biomedical applications. Nanoscale.

[163] Kumar S., Parekh S.H. (2021). Molecular control of interfacial fibronectin structure on graphene oxide steers cell fate. ACS Appl Mater Interfaces.

[164] Tavafoghi M., Brodusch N., Gauvin R., Cerruti M. (2016). Hydroxyapatite formation on graphene oxide modified with amino acids: arginine versus glutamic acid. J R Soc Interface.

[165] Subbiah R., Du P., Van S.Y., Suhaeri M., Hwang M.P., Lee K. (2014). Fibronectin-tethered graphene oxide as an artificial matrix for osteogenesis. Biomed Mater.

[166] Kang E.S., Song I., Kim D.S., Lee U., Kim J.K., Son H. (2018). Size-dependent effects of graphene oxide on the osteogenesis of human adipose-derived mesenchymal stem cells. Colloid Surf B-Biointerfaces.

[167] Li W.C., Su Z.N., Hu Y.R., Meng L.H., Zhu F., Xie B. (2024). Functional and structural construction of photothermal-responsive PEEK composite implants to promote bone regeneration and bone-implant integration. Compos Sci Technol.

[168] Xue D.T., Chen E.M., Zhong H.M., Zhang W., Wang S.D., Joomun M.U. (2018). Immunomodulatory properties of graphene oxide for osteogenesis and angiogenesis. Int J Nanomed.

[169] Sun J.C., Li L., Xing F., Yang Y., Gong M., Liu G.M. (2021). Graphene oxide-modified silk fibroin/nanohydroxyapatite scaffold loaded with urine-derived stem cells for immunomodulation and bone regeneration. Stem Cell Res Ther.

[170] Li Q.F., Shen A.F., Wang Z.L. (2020). Enhanced osteogenic differentiation of BMSCs and M2-phenotype polarization of macrophages on a titanium surface modified with graphene oxide for potential implant applications. RSC Adv.

[171] Zhang Z.Y., Shao J.X., Gao Y., Li Y.H., Liu T., Yang M.D. (2023). Research progress and future prospects of antimicrobial modified polyetheretherketone (PEEK) for the treatment of bone infections. Front Bioeng Biotechnol.

[172] Yu C.H., Chen G.Y., Xia M.Y., Xie Y., Chi Y.Q., He Z.Y. (2020). Understanding the sheet size-antibacterial activity relationship of graphene oxide and the nano-bio interaction-based physical mechanisms. Colloid Surf B-Biointerfaces.

[173] Liu S., Zeng T.H., Hofmann M., Burcombe E., Wei J., Jiang R. (2011). Antibacterial activity of graphite, graphite oxide, graphene oxide, and reduced graphene oxide: membrane and oxidative stress. ACS Nano.

[174] Huang J., Zhang D., Zhu C., Chen S., Wang Y., Han K. (2025). Graphene-based nanomaterials: mechanisms and potentials in the fight against multidrug resistant bacterial infections: a review. RSC Adv.

[175] Linklater D.P., Baulin V.A., Juodkazis S., Ivanova E.P. (2018). Mechano-bactericidal mechanism of graphene nanomaterials. Interface Focus.

[176] Chen Y.Q., Wu W., Xu Z.Q., Jiang C., Han S., Ruan J. (2020). Photothermal-assisted antibacterial application of graphene oxide-Ag nanocomposites against clinically isolated multi-drug resistant Escherichia coli. R Soc Open Sci.

[177] Wu J., Wang C.M., Zhang S.S., Zhang L., Hao J.S., Jia Z.J. (2024). Preparation and properties of GO/ZnO/nHAp composite microsphere bone regeneration material. Micromachines.

[178] Wang J.H., Wang Y.Y., Liu D.S., Yang Q., Huang C.G., Yang C.R. (2018). Preparation and cytological study of collagen/nano-hydroxyapatite/graphene oxide composites. Acta Bioeng Biomech.

[179] Shao W., Liu X.F., Min H.H., Dong G.H., Feng Q.Y., Zuo S.L. (2015). Preparation, characterization, and antibacterial activity of silver nanoparticle-decorated graphene oxide nanocomposite. ACS Appl Mater Interfaces.

[180] Li B.Y., Gao X.X., Qu J.G., Xiong F., Xuan H.Y., Jin Y. (2022). Visible-light-driven antimicrobial activity and mechanism of polydopamine-reduced graphene Oxide/BiVO4 composite. Int J Mol Sci.

[181] Liao C., Li Y., Tjong S.C. (2018). Graphene nanomaterials: synthesis, biocompatibility, and cytotoxicity. Int J Mol Sci.

[182] Zheng J., Zhao H., Dong E., Kang J., Liu C., Sun C. (2021). Additively-manufactured PEEK/HA porous scaffolds with highly-controllable mechanical properties and excellent biocompatibility. Mater Sci Eng C Mater Biol Appl.

[183] Kang E.S., Kim D.S., Han Y., Son H., Chung Y.H., Min J. (2018). Three-dimensional Graphene-RGD peptide nanoisland composites that enhance the osteogenesis of human adipose-derived mesenchymal stem cells. Int J Mol Sci.

[184] Wang W., Liu Y., Yang C., Qi X., Li S.W., Liu C.S. (2019). Mesoporous bioactive glass combined with graphene oxide scaffolds for bone repair. Int J Biol Sci.

[185] Su Z., Zhang J., Tan P.J., Zhu S.S., Jiang N. (2022). Selective polyetheretherketone implants combined with graphene cause definitive cell adhesion and osteogenic differentiation. Int J Nanomed.

[186] Zhang Q.R., Zhang C.D. (2020). Chronic exposure to low concentration of graphene oxide increases bacterial pathogenicity via the envelope stress response. Environ Sci Technol.

[187] Iravani M., Simjoo M., Chahardowli M., Moghaddam A.R. (2024). Experimental insights into the stability of graphene oxide nanosheet and polymer hybrid coupled by ANOVA statistical analysis. Sci Rep.

[188] Khalil W.A., Sherif H.H.A., Hemdan B.A., Khalil S.K.H., Hotaby W.E.I. (2019). Biocompatibility enhancement of graphene oxide-silver nanocomposite by functionalisation with polyvinylpyrrolidone. IET Nanobiotechnol.

[189] Zheng J., Zhao H., Ouyang Z., Zhou X., Kang J., Yang C. (2022). Additively-manufactured PEEK/HA porous scaffolds with excellent osteogenesis for bone tissue repairing. Compos Pt B-Eng.

[190] Cheng B.C., Jaffee S., Averick S., Swink I., Horvath S., Zhukauskas R. (2020). A comparative study of three biomaterials in an ovine bone defect model. Spine J.

[191] Baheti W., Chen X.T., La M., He H.Y. (2024). Biomimetic HA-GO implant coating for enhanced osseointegration via macrophage M2 polarization-induced osteo-immunomodulation. J Appl Biomater Funct Mater.

[192] Sun C., Kang J., Yang C., Zheng J., Su Y., Dong E. (2022). Additive manufactured polyether-ether-ketone implants for orthopaedic applications: a narrative review. Biomaterials Translational.

[193] Wang C.C., Hu H.X., Li Z.P., Shen Y.F., Xu Y., Zhang G.Q. (2019). Enhanced osseointegration of titanium alloy implants with laser microgrooved surfaces and graphene oxide coating. ACS Appl Mater Interfaces.

[194] Li H., Buck E., Elkashty O., Tran S.D., Szkopek T., Cerruti M. (2022). Graphene oxide/elastin nanostructure-based membranes for bone regeneration. ACS Appl Nano Mater.

[195] Liang J.Q., Liu P.L., Yang X.Q., Liu L., Zhang Y., Wang Q. (2023). Biomaterial-based scaffolds in promotion of cartilage regeneration: recent advances and emerging applications. J Orthop Transl.

[196] Uysal I., Tezcaner A., Evis Z. (2024). Methods to improve antibacterial properties of PEEK: a review. Biomed Mater.

[197] AlOtaibi N., Naudi K., Conway D., Ayoub A. (2020). The current state of PEEK implant osseointegration and future perspectives: a systematic review. Eur Cell Mater.

[198] Inchingolo F., Inchingolo A.M., Latini G., Palmieri G., Di Pede C., Trilli I. (2023). Application of graphene oxide in oral surgery: a systematic review. Materials.

[199] Wong K.C., Sze L.K.Y., Kumta S.M. (2021). Complex joint-preserving bone tumor resection and reconstruction using computer navigation and 3D-printed patient-specific guides: a technical note of three cases. J Orthop Transl.

[200] Mazinani A., Rastin H., Nine M.J., Lee J., Tikhomirova A., Tung T.T. (2021). Comparative antibacterial activity of 2D materials coated on porous-titania. J Mat Chem B.

[201] Lu S.L., Wang Y.H., Liu G.F., Wang L., Li Y., Guo Z.Y. (2021). Graphene oxide nanoparticle-loaded ginsenoside Rg3 improves photodynamic therapy in inhibiting malignant progression and stemness of Osteosarcoma. Front Mol Biosci.

[202] Zhang Y.L., Zhai D., Xu M.C., Yao Q.Q., Chang J., Wu C.T. (2016). 3D-printed bioceramic scaffolds with a Fe3O4/graphene oxide nanocomposite interface for hyperthermia therapy of bone tumor cells. J Mat Chem B.

[203] Laurenti M., Lamberti A., Genchi G.G., Roppolo I., Canavese G., Vitale-Brovarone C. (2019). Graphene oxide finely tunes the bioactivity and drug delivery of mesoporous ZnO scaffolds. ACS Appl Mater Interfaces.

[204] Boran G., Tavakoli S., Dierking I., Kamali A.R., Ege D. (2020). Synergistic effect of graphene oxide and zoledronic acid for osteoporosis and cancer treatment. Sci Rep.

[205] Yao L., Dong W., Zhang M., Jiang Z., Dong W. (2025). Elevated photothermal antibacterial efficacy and improved biocompatibility via photothermal cryogel modification for carbon fiber‐reinforced polyether ether ketone composites. Polym Compos.

[206] Liao P., Tong S.H., Du L., Mei J., Wang B.Q., Lu Y.F. (2025). Single-cell transcriptomics identifies the common perturbations of monocyte/macrophage lineage cells in inflammaging of bone marrow. J Orthop Transl.

[207] Makino T., Takaneka S., Sakai Y., Yoshikawa H., Kaito T. (2021). Impact of mechanical stability on the progress of bone ongrowth on the frame surfaces of a titanium-coated PEEK cage and a 3D porous titanium alloy cage: in vivo analysis using CT color mapping. Eur Spine J.

[208] de Almeida M.V.R., Ribeiro M.C.O., dos Reis-Neta G.R., Vargas-Moreno V.F., Gomes R.S., da Silva W.J. (2024). Dental implant and abutment in PEEK: stress assessment in single crown retainers on anterior region. Clin Oral Investig.

[209] Yuan K., Zhang K., Yang Y.Q., Lin Y.X., Zhou F., Mei J.T. (2022). Evaluation of interbody fusion efficacy and biocompatibility of a polyetheretherketone/calcium silicate/porous tantalum cage in a goat model. J Orthop Transl.

[210] Yang Y., Li M., Luo H., Zhang D. (2022). Surface-decorated graphene oxide sheets with copper nanoderivatives for bone regeneration: an in vitro and in vivo study regarding molecular mechanisms, osteogenesis, and anti-infection potential. ACS Infect Dis.

[211] Ricci A., Cataldi A., Zara S., Gallorini M. (2022). Graphene-oxide-enriched biomaterials: a focus on osteo and chondroinductive properties and immunomodulation. Materials.

[212] Mukherjee S.P., Gliga A.R., Lazzaretto B., Brandner B., Fielden M., Vogt C. (2018). Graphene oxide is degraded by neutrophils and the degradation products are non-genotoxic. Nanoscale.

[213] Yadav S., Singh Raman A.P., Meena H., Goswami A.G., Bhawna Kumar V. (2022). An update on graphene oxide: applications and toxicity. ACS Omega.

[214] Pahlevanzadeh F., Emadi R., Setayeshmehr M., Kharaziha M., Poursamar S.A. (2022). Antibacterial amorphous magnesium phosphate/graphene oxide for accelerating bone regeneration. Biomater Adv.

[215] Achawi S., Feneon B., Pourchez J., Forest V. (2021). Structure-activity relationship of graphene-based materials: impact of the surface chemistry, surface specific area and lateral size on their in vitro toxicity. Nanomaterials.

[216] Zancanela D.C., Simao A.M.S., Francisco C.G., de Faria A.N., Ramos A.P., Gonçalves R.R. (2016). Graphene oxide and titanium: synergistic effects on the biomineralization ability of osteoblast cultures. J Mater Sci Mater Med.

[217] Yang Y., Li M., Zhou B.X., Jiang X.L., Zhang D., Luo H. (2023). Graphene oxide/gallium nanoderivative as a multifunctional modulator of osteoblastogenesis and osteoclastogenesis for the synergistic therapy of implant-related bone infection. Bioact Mater.

[218] Xu C., Ma B., Peng J.L., Gao L., Xu Y.H., Huan Z.G. (2019). Tricalcium silicate/graphene oxide bone cement with photothermal properties for tumor ablation. J Mat Chem B.

[219] Li Y.Z., Yang L., Hou Y., Zhang Z.Z., Chen M., Wang M.X. (2022). Polydopamine-mediated graphene oxide and nanohydroxyapatite-incorporated conductive scaffold with an immunomodulatory ability accelerates periodontal bone regeneration in diabetes. Bioact Mater.

[220] Ma S.Q., Wang C.W., Dong Y.F., Jing W., Wei P.F., Peng C. (2022). Microsphere-gel composite system with mesenchymal stem cell recruitment, antibacterial, and immunomodulatory properties promote bone regeneration via sequential release of LL37 and W9 peptides. ACS Appl Mater Interfaces.

[221] Wu S.D., Shuai Y., Qian G.W., Peng S.P., Liu Z., Shuai C.J. (2023). A spatiotemporal drug release scaffold with antibiosis and bone regeneration for osteomyelitis. J Adv Res.

[222] Zhang M., Matinlinna J.P., Tsoi J.K.H., Liu W., Cui X., Lu W.W. (2020). Recent developments in biomaterials for long-bone segmental defect reconstruction: a narrative overview. J Orthop Translat.

[223] Zhang Z.J., Wang Y.K., Teng W.S.Y., Zhou X.Z., Ye Y.X., Zhou H. (2021). An orthobiologics-free strategy for synergistic photocatalytic antibacterial and osseointegration. Biomaterials.

[224] Xing J., Liu S. (2024). Application of loaded graphene oxide biomaterials in the repair and treatment of bone defects. Bone Jt Res.

[225] Jang H.J., Kang M.S., Kim W.H., Jo H.J., Lee S.H., Hahm E.J. (2023). 3D printed membranes of polylactic acid and graphene oxide for guided bone regeneration. Nanoscale Adv.

[226] Ligorio C., Zhou M., Wychowaniec J.K., Zhu X.Y., Bartlam C., Miller A.F. (2019). Graphene oxide containing self-assembling peptide hybrid hydrogels as a potential 3D injectable cell delivery platform for intervertebral disc repair applications. Acta Biomater.

[227] Zhang Y., Yu W., Wang J., Zhan T., Kamran M.A., Wang K. (2023). Long-term exposure of graphene oxide suspension to air leading to spontaneous radical-driven degradation. Environ Sci Technol.

[228] Lalwani G., D'Agati M., Khan A.M., Sitharaman B. (2016). Toxicology of graphene-based nanomaterials. Adv Drug Deliv Rev.

[229] Yang K., Wu Z., Zhang K., Weir M.D., Xu H.H.K., Cheng L. (2024). Unlocking the potential of stimuli-responsive biomaterials for bone regeneration. Front Pharmacol.

[230] Hua S., de Matos M.B.C., Metselaar J.M., Storm G. (2018). Current trends and challenges in the clinical translation of nanoparticulate nanomedicines: pathways for translational development and commercialization. Front Pharmacol.

[231] Herczeg C.K., Song J. (2022). Sterilization of polymeric implants: challenges and opportunities. ACS Appl Bio Mater.

[232] El-Yamany N.A., Mohamed F.F., Salaheldin T.A., Tohamy A.A., Abd El-Mohsen W.N., Amin A.S. (2017). Graphene oxide nanosheets induced genotoxicity and pulmonary injury in mice. Exp Toxicol Pathol.

[233] Zheng Y., Gao A., Bai J., Liao Q., Wu Y., Zhang W. (2022). A programmed surface on polyetheretherketone for sequentially dictating osteoimmunomodulation and bone regeneration to achieve ameliorative osseointegration under osteoporotic conditions. Bioact Mater.

[234] Shi L., Xuan D., Jakovljevic M. (2024). A review on the evolving environment of medical device real-world evidence regulation on market access in the USA. Cost Eff Resour Alloc.

[235] Akmal J.S., Salmi M., Mäkitie A., Björkstrand R., Partanen J. (2018). Implementation of industrial additive manufacturing: intelligent implants and drug delivery systems. J Func Biomater.

